# In Situ XRPD Investigation of Relative Humidity-Induced Lattice Responses in Tetragonal Hen Egg-White Lysozyme

**DOI:** 10.3390/biom16030442

**Published:** 2026-03-15

**Authors:** Marios Konstantopoulos, Stamatina Kafetzi, Dimitrios Koutoulas, Christina Papaefthymiou, Marianna Lampropoulou, Theodora Alexiou, Maria Nefeli Karagrigoriou, Nikolaos Pagonis, Artemis Karapeti, Angelos Kontarinis, Detlef Beckers, Thomas Degen, Irene Margiolaki

**Affiliations:** 1 Department of Biology, Section of Genetics, Cell Biology and Development, University of Patras, 26500 Patras, Greece; mariosk2001@gmail.com (M.K.); matkaf1999@gmail.com (S.K.); koutoulasjim5@gmail.com (D.K.); xristina.papaefthimiou@gmail.com (C.P.); marianna.lampropoulou@gmail.com (M.L.); theodora.alexiou5@gmail.com (T.A.); marianefeli33@gmail.com (M.N.K.); pagonisnikos014@gmail.com (N.P.); artemis.karapeti@gmail.com (A.K.); kontarinisangelos@gmail.com (A.K.); 2 European Synchrotron Radiation Facility, CS40220, 38043 Grenoble Cedex 9, Rhônes-Alpes, France; 3 Malvern Panalytical B.V., Lewyleg 1, 7602 EA Almelo, The Netherlands; detlef.beckers@malvernpanalytical.com (D.B.); thomas.degen@malvernpanalytical.com (T.D.)

**Keywords:** X-ray crystallography, protein crystallography, hen egg-white lysozyme, humidity variation, in situ X-ray powder diffraction

## Abstract

Protein crystals are intrinsically hydrated systems, and their structural integrity is strongly influenced by environmental humidity. Understanding the effects of relative humidity (RH) variation on crystal stability is therefore essential for both fundamental research and applied studies. In this work, the structural response of tetragonal hen egg-white lysozyme (HEWL) to controlled RH variation was investigated using in situ X-ray powder diffraction (XRPD). Polycrystalline HEWL samples were subjected to systematic gradual dehydration and rehydration cycles, as well as to non-gradual RH variation protocols. Pawley analysis of the XRPD data enabled monitoring of the evolution of unit cell parameters and unit cell volume as a function of RH. Under all experimental conditions, the tetragonal polymorph (space group *P*4_3_2_1_2; *a* = 79.105 (4) Å, *c* = 38.231 (2) Å) was preserved. RH variation induced smooth, continuous and anisotropic lattice changes, characterized by a decrease in the *a* (=*b*)-axis and a concomitant increase in the *c*-axis upon dehydration, while rehydration resulted in the opposite behavior. The overall magnitude of lattice variation remained limited (within ±2%), indicating a high degree of structural stability. Partial degradation of crystallinity was observed only after prolonged exposure to low RH levels. These findings demonstrate the remarkable structural resilience of tetragonal HEWL and highlight the effectiveness of in situ XRPD as a powerful tool for probing hydration-driven lattice responses in protein crystals under realistic environmental conditions.

## 1. Introduction

Proteins adopt their intrinsic conformation and perform their physiological function in aqueous solutions, emphasizing the importance of hydration for protein stability, structure and dynamics [1]. Water molecules participate actively in stabilizing secondary and tertiary structural elements, mediating intermolecular interactions and enabling conformational flexibility essential for biological activity. As a result, variations in environmental conditions such as relative humidity (RH) and temperature can directly influence protein structure and functionality. Systematic investigation of hydration-driven structural effects has therefore gained increasing attention in structural biology, particularly in the context of X-ray crystallography, where protein crystals represent highly ordered yet intrinsically hydrated systems.

Protein crystals are distinguished by their exceptionally high solvent content, typically ranging from 30% to 70% of the crystal volume, distributed within extended solvent channels permeating the lattice [2,3]. This hydration architecture leads to relatively loose molecular packing stabilized predominantly by weak, water-mediated intermolecular interactions, including hydrogen bond networks and van der Waals forces [4,5]. While this extensive solvent network preserves the native structure of the protein, it also renders protein crystals particularly sensitive to environmental disturbances. Changes in RH can alter both the amount and spatial organization of solvent within the lattice, inducing measurable variations in unit cell parameters, intermolecular spacing and crystal packing, and in some cases leading to a loss of crystalline order.

The relationship between hydration state and crystallographic behavior has been recognized since early dehydration experiments demonstrated that complete solvent removal results in crystal collapse and loss of the diffraction signal [6,7]. Subsequent studies showed that controlled gradual modification of RH can reversibly tune the solvent content of protein crystals, often improving diffraction quality while preserving crystallinity [8,9,10,11]. These observations establish dehydration and rehydration as powerful post-crystallization treatments, complementing optimization of crystallization conditions and cryoprotection strategies [12,13]. Importantly, such processes involve diffusive transport of water within the crystal, which can induce isotropic lattice responses and molecular rearrangements [14,15].

While single-crystal X-ray diffraction (SCXRD) remains the method of choice for atomic resolution structural determination, it presents practical limitations when investigating dynamic or non-equilibrium environmental conditions. Single crystals are often fragile, prone to cracking or loss of diffraction quality during dehydration and rehydration cycles and may not be representative of the bulk material, particularly in systems comprising microcrystalline aggregates. This limitation is especially relevant in pharmaceutical and biotechnological contexts, where many products consist of polycrystalline or microcrystalline protein formulations produced via batch crystallization methods [16,17].

In this framework, X-ray powder diffraction (XRPD) has emerged as a powerful complementary technique for studying protein crystals as ensembles of microcrystals rather than individual specimens, and its methodological development and broad applicability have been extensively reviewed [18,19]. Advances in instrumentation [20], data acquisition and analysis methodologies [21,22] have enabled the collection of high-quality XRPD data from biological macromolecules, allowing phase identification [23,24,25,26,27,28,29,30,31], polymorph screening and structure refinement and solution [32,33,34,35,36,37]. Macromolecular powder diffraction has been successfully applied to a wide range of protein and peptide systems of pharmaceutical relevance, enabling the study of structural responses to environmental variables such as RH [38,39,40,41], temperature and chemical composition [22,25,26,27,30,31,42]. Recent developments in humidity- and temperature-controlled sample environments have further expanded the capabilities of in situ XRPD. Modern humidity chambers allow precise, continuous and reversible control of RH and temperature, overcoming the limitations of earlier approaches based on saturated salt solutions which provided only discrete RH values and limited temporal control [43,44]. These advances enable real-time monitoring of lattice evolution under both near-equilibrium and non-equilibrium conditions, capturing transient structural states that are inaccessible to static measurements [21,22].

The protein studied in this case, hen egg-white lysozyme (HEWL), is widely studied and used as a model protein in structural biology [39,45,46,47,48,49,50,51,52,53,54,55,56]. HEWL is a small biomolecule (14 kDa), exhibiting strong antibacterial activity. Its action is dual in nature, functioning enzymatically by hydrolysing *β*-1,4-glycosidic bonds of peptidoglycan in bacterial cell walls and, as a cationic protein, disrupting membrane integrity and inducing cell wall rearrangement via autolytic enzymes [57]. In addition to its bacteriolytic activity, lysozyme encompasses an immune-regulatory role by inducing cytokine release and host inflammatory responses [58]. The combination of these functions highlights its significance in contemporary pharmaceutical research.

Previous XRPD studies on HEWL polycrystalline samples have demonstrated systematic and anisotropic lattice responses to controlled RH variation, revealing continuous evolution of unit cell parameters and identifying critical RH thresholds associated with loss of crystallinity [38,39]. These studies highlight the importance of equilibration time and polymorph-specific hydration architecture in determining crystal stability. However, comprehensive comparison of gradual versus non-gradual relative humidity variation protocols, combined with systematic reproducibility analysis and repeated cycling experiments, remains limited, particularly for the tetragonal lysozyme polymorph. The present study addresses this gap by employing in situ XRPD measurements under precisely controlled RH. By subjecting tetragonal HEWL polycrystalline samples to both gradual and non-gradual dehydration and rehydration protocols including repeated RH cycling, this work aims to elucidate the lattice-level structural response, reproducibility and tolerance of the system under realistic environmental stress conditions. Through quantitative analysis of unit cell parameters and volume as a function of RH, this study advances understanding of hydration-driven structural behavior in protein crystals and further demonstrates the methodological value of in situ XRPD for probing protein and environment interactions in polycrystalline materials.

## 2. Materials and Methods

### 2.1. Crystallization

**Figure 1:**
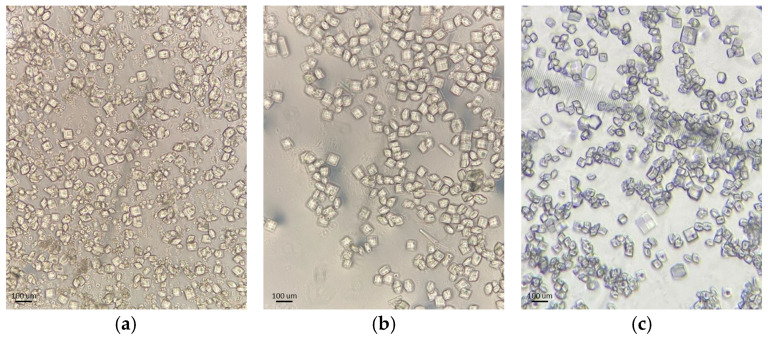
Optical microscopy images of representative HEWL polycrystalline samples: (**a**) Series 3, (**b**) Series 4 and (**c**) Series 5.

Lyophilized HEWL was purchased from AppliChem (product No. A4972; CAS-No. 9001-63-2). The crystallization protocol was based on previously published methodologies [14,39], which were lightly altered to optimize the production of the polycrystalline samples. HEWL was crystallized by utilizing the batch technique and specifically the salting-out method [59]. This methodology was performed five times in total, resulting in five experimental series, in order to confirm the reproducibility of our results. A crystallization series is defined as a continuous experimental sequence in which a single polycrystalline HEWL sample is subjected to a predefined sequence of RH variations. The process commenced with the preparation of the protein solution. Stock solution of HEWL at 200 mg mL^−1^ was prepared in a 1.1 mL volume by dissolving 220 mg of lyophilized HEWL in double-distilled water (ddH_2_O). Subsequently, stock solutions of 5 *M* sodium chloride (Ing. Petr Švec – PENTA s.r.o., Prague, Czech Republic), 60% *w/v* polyethylene glycol 6000 (AppliChem GmbH, Darmstadt, Germany) and 1 *M* sodium acetate (FERAK Berlin GmbH, Berlin, Germany) were prepared. To produce the precipitation solution, 0.9 mL of 5 *M* sodium chloride, 0.5 mL of 60% *w/v* polyethylene glycol 6000, 0.075 mL of 1 *M* sodium acetate and 0.025 mL of ddH_2_O were mixed, resulting in 1.5 mL of the precipitation solution. For the preparation of four polycrystalline samples, 0.25 mL of the protein solution was mixed with 0.25 mL of the precipitation solution, resulting in a volume of 0.5 mL per sample. The final concentration of HEWL in the polycrystalline samples was 100 mg mL^−1^ (Table 1). Crystals were grown at room temperature (~298 K) and after 48 h each of the solutions yielded ~50 μL of the polycrystalline sample (Figure 1).

### 2.2. XRPD Data Collection Under Ambient Conditions

Initial X-ray powder diffraction (XRPD) data were collected under ambient conditions for the structural characterization of the polycrystalline samples. Collection was conducted using the laboratory X’Pert Pro diffractometer (Malvern Panalytical, Almelo, The Netherlands), exploiting Debye–Scherrer geometry. X-rays were generated using a Cu anode operated at 40 kV and 45 mA, providing Cu Kα radiation [λ = 1.540585(3) Å] [60] at RT (room temperature).

Polycrystalline samples were loaded into borosilicate glass capillaries of 1.0 mm inner diameter. Prior to data collection, the capillaries were centrifuged to achieve dense and homogenous packing of the crystalline material in the incident beam path, while excess mother liquor was carefully removed. The capillaries were subsequently sealed with silicon vacuum grease to prevent solvent evaporation and mounted on the goniometer head of a translating capillary spinner.

On the incident beam path, Cu K*α* radiation was conditioned using a focusing X-ray mirror combined with a 0.5° anti-scatter slit, a 0.04 rad Soller slit, a 10 mm beam mask and a 0.5° divergence slit. On the diffracted beam path, a 0.04 rad Soller slit and a PIXcel^1D^ detector equipped with anti-scatter shielding were employed. Data acquisition was performed starting from 2θ = 0°, with detector protection ensured by a beam stop mounted on the anti-scatter assembly of the X-ray mirror.

Diffraction data were collected at a fixed capillary position over a 2θ range of 0–30° through multiple and consecutive measurements. Individual scans had a duration of approximately 30 min. A total of 25 scans were acquired that were summed up subsequently to enhance counting statistics and improve the signal-to-noise ratio. No indications of radiation damage were detected over the total acquisition time of approximately 12.5 h.

### 2.3. In Situ XRPD Data Collection

In situ XRPD measurements were performed under controlled RH conditions using the laboratory X’Pert Pro diffractometer equipped with a transmission temperature–humidity chamber, specifically the Multi-sample Humidity Chamber-trans (MHC-trans, Anton Paar) [61]. The incident beam was conditioned using a focusing X-ray mirror for Cu Kα radiation [λ = 1.540585(3) Å], together with a 0.25° divergence slit, 0.25° anti-scatter slit, and 0.04 rad Soller slit, resulting in an incident beam size of approximately 0.4–0.5 mm at the sample position. The diffracted beam was collected using a PIXcel1D detector equipped with anti-scatter shielding, a 7.5 mm anti-scatter slit, and a 0.04 rad Soller slit. The effective illuminated sample volume was limited by the 1 mm capillary diameter. Diffraction data were collected over a 2θ angular range of approximately 1.2–30°, as the presence of the beam stop and the geometry of the humidity chamber do not permit reliable signal detection at lower diffraction angles. This angular range corresponds to a minimum d-spacing of approximately 3.0 Å and was sufficient for reliable extraction of lattice parameters via Pawley refinement and FR monitoring RH-induced structural variations. All measurements were carried out at a stable temperature of 294.15 K.

Polycrystalline precipitates of HEWL were recovered from Eppendorf tubes by centrifugation, and excess mother liquor was carefully removed. The remaining crystalline material was gently transferred onto dedicated sample holders of a 12 mm diameter, covered with Kapton foil of 125 μm thickness, in order to minimize background contributions during data collection. During sample preparation, particular care was taken to minimize mechanical stress on the crystals, while a small amount of residual mother liquor was retained to prevent the degradation of the crystalline sample. The sample holders were mounted on the rotating sample changer, which allows the load of up to eight samples at the same time, inside the humidity chamber, allowing sequential in situ measurements under varying environmental conditions.

**Figure 2:**
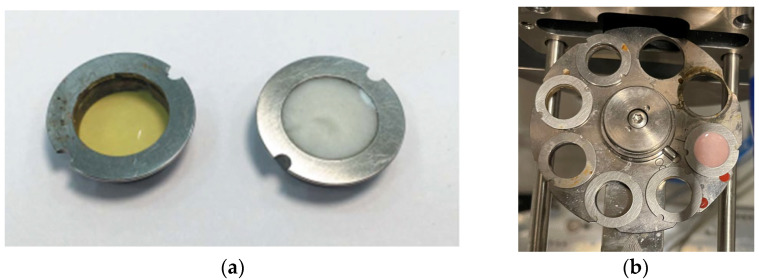

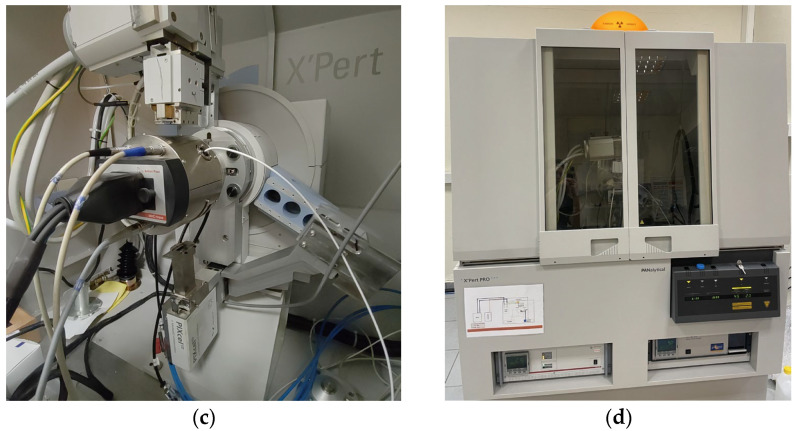
Components of the in situ XRPD experimental setup: (**a**) an empty [**left**] and a loaded with polycrystalline HEWL sample [**right**] Kapton holder, (**b**) the multi-position rotor, (**c**) the humidity chamber MHC-trans mounted on the diffractometer, where dehydration and rehydration are achieved through controlled mixing of dry and humidified air streams at a defined flow rate and constant temperature, and (**d**) external view of the laboratory diffractometer with the MHG-32 and TCU-60M control units visible on its lower side.

The temperature–humidity chamber provides independent control of RH and temperature through external humidity and temperature control units, the MHG-32 (Modular Humidity Generator) and TCU-60M (Temperature Control Unit) respectively. The control units enable RH regulation in the range of 5–95% at temperatures between 10 °C and 60 °C. Full and stable RH control is achieved at temperatures between 20 °C and 60 °C. The required environment inside the chamber was established using dry air supplied by an air compressor with a regulated flow rate of up to 250 mL/min, while the temperature of the chamber enclosure was stabilized by a water circulator in order to prevent overheating. The stability of the experimental conditions was ensured by high-precision sensors (Figure 2).

The in situ XRPD experiments were conducted using predefined RH modulation protocols. These included both gradual dehydration–rehydration cycles and non-gradual RH variation cycles, designed to probe lattice evolution under different RH change rates. In gradual experiments, RH was changed stepwise through a series of intermediate rates levels, allowing the crystal lattice to equilibrate progressively at its state. In contrast, non-gradual experiments involved rapid changes between selected arrays values without intermediate equilibration steps. This distinction allows assessment of whether the structural response of the crystal lattice depends on the rate at which hydration conditions are modified. Each experimental cycle followed a common sequence: (i) setting the target RH level, (ii) allowing the system to equilibrate for a predefined waiting time and (iii) collecting XRPD data. The RH was subsequently adjusted according to the selected protocol, and this procedure was repeated until completion of the cycle.

Series 1 and Series 2 were designed to investigate gradual dehydration and rehydration behavior. In Series 1, a single cycle was performed in which the RH was decreased stepwise from 95% to 75% using uniform 5% RH steps and subsequently followed by rehydration back to 95% using the same step size. At each RH level, the system was allowed to equilibrate for 120 min prior to data collection, and 20 individual scans were collected per RH level. In Series 2, two gradual dehydration–rehydration cycles were carried out over an extended RH range, from 95% down to 67% and back to 95%. The RH variation employed a combination of step sizes (5%, 3% and 2%) in order to provide finer sampling in selected humidity regions. For both cycles, a constant equilibration time of 60 min was applied at each RH level. During the first cycle, 10 scans were collected per RH level, while in the second cycle the number of scans per RH level was reduced to 5, in order to limit total exposure time while preserving sufficient counting statistics.

**Figure 3:**
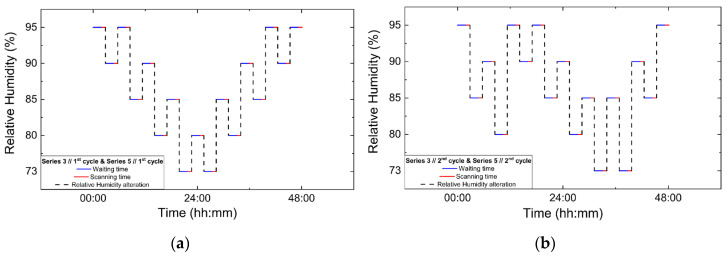

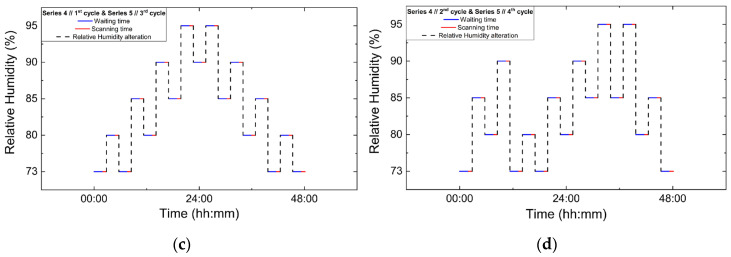
Experimental timelines of the in situ XRPD data collection protocols under controlled RH variations. Panels show paired non-gradual RH variation cycles: (**a**) Series 3, Cycle 1 and its repetition in Series 5, Cycle 1; (**b**) Series 3, Cycle 2 and Series 5, Cycle 2; (**c**) Series 4, Cycle 1 and Series 5, Cycle 3; and (**d**) Series 4, Cycle 2 and Series 5, Cycle 4. Blue lines indicate the waiting (equilibration) time at each RH level, red lines correspond to XRPD data collection periods and black dashed lines represent the imposed RH variation profile.

Series 3, Series 4 and Series 5 focused on non-gradual RH variation protocols, involving successive increases and decreases in RH rather than dehydration–rehydration sequences. In Series 3 and Series 4, two consecutive non-gradual cycles were performed in each series. RH changes were implemented using step sizes of 7% and 5%, with the system equilibrated for 120 min at each RH level prior to data acquisition. For each RH level 5 XRPD scans were collected. Series 5 comprised the most extensive set of non-gradual RH variation experiments. Four consecutive non-gradual cycles were initially performed using RH step sizes of 7% and 5%, with a fixed equilibration time of 120 min and 5 scans collected per RH level. Furthermore, these cycles were designed to replicate the non-gradual RH variation protocols employed in Series 3 and Series 4. These experiments were followed by three additional cycles employing progressively broader RH ranges. Specifically, one gradual dehydration–rehydration cycle was carried out between 95% and 75% RH using 5% steps, followed by a cycle spanning from 95% down to 51% RH and back to 95%, employing variable step sizes of 5%, 4%, 2% and 1%. Finally, a cycle with an extended range was performed between 95% and 30% RH using RH steps of 10%, 5% and 2%. For these latter cycles, the equilibration time was set to 120 min, except for the lowest RH cycle (down to 30% RH), where a reduced waiting time of 90 min was adopted in order to limit total experimental duration. In all cycles of Series 5, 5 scans were collected at each RH level. The protocols for data collection under non-gradual RH variations are visually represented in Figure 3, while the overall experimental flow is represented in Table 2.

### 2.4. XRPD Data Treatment and Analysis

Following data collection, all laboratory in situ XRPD patterns were analyzed using the Pawley refinement method [62] as implemented in the crystallographic software HighScore Plus [63]. Prior to refinement, the individual diffraction scans collected at each RH level were not treated independently. Instead, all scans corresponding to the same RH level were systematically compared using overplot functionality in HighScore Plus (Version: 5.3.1) and WinPlotR (Version: Octorber 2023) [64]. Scans exhibiting identical peak positions, comparable intensity distributions and showing no evidence of peak shifts or single-crystal spikes were merged to generate a representative diffraction pattern for each RH level.

The resulting merged XRPD profiles were subsequently subjected to Pawley fitting, enabling the accurate determination of unit cell parameters as well as the refinement of peak shape parameters and background coefficients. This approach allowed reliable monitoring of subtle changes in unit cell parameters and unit cell volume as a function of RH. The extracted unit cell parameters provided a direct measure of the structural response of HEWL crystals to both gradual and non-gradual RH variations, facilitating a comparative analysis of lattice evolution under different conditions.

## 3. Results

### 3.1. Phase Identification via Capillary XRPD Measurements

**Figure 4:**
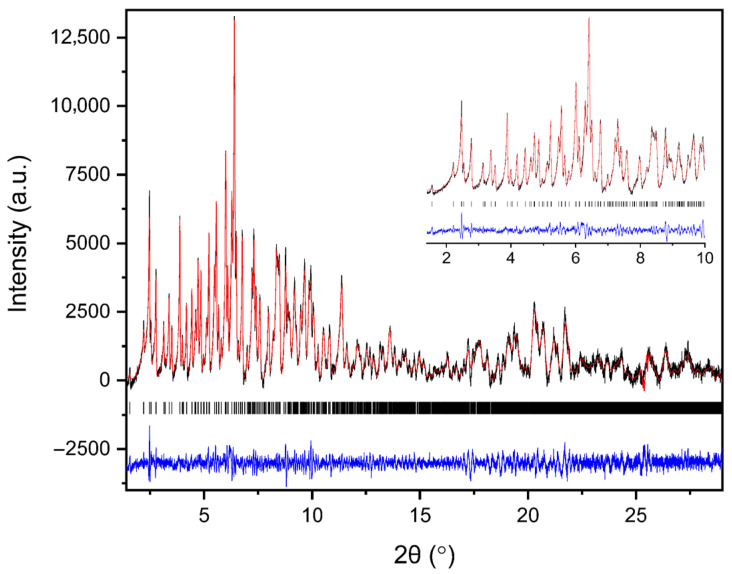
Pawley fit of a representative single-capillary XRPD pattern of HEWL. Black line represents the observed data, red line represents the calculated profile, and blue line shows the difference between the calculated and experimental data. The black vertical tick marks depict the Bragg reflection positions corresponding to the space group *P*4_3_2_1_2 of tetragonal symmetry [*a* = 79.105(4) Å, *c* = 38.231(2) Å, with agreement factors *R_wp_* = 1.37% and *χ*^2^ = 1.98].

Polycrystalline samples obtained from all crystallization series were initially characterized by XRPD measurements collected in capillary geometry under ambient conditions, with one capillary measured for each crystallization series. The diffraction patterns revealed a single crystalline phase, with no evidence of phase coexistence. Indexing and subsequent Pawley analysis confirmed that the material crystallizes in the tetragonal space group *P*4_3_2_1_2. The refined unit cell parameters were consistent across all capillary datasets (five in total), indicating good reproducibility of the crystallization procedure. A representative Pawley fit from the capillary measurements is shown in Figure 4. Pawley analysis of the laboratory XRPD data yielded satisfactory results. Lattice parameters were determined as *a* = 79.105(4) Å, *c* = 38.231(2) Å, with agreement factors *R_wp_* = 1.3749% and *χ^2^* = 1.98421.

Results demonstrate the high quality of the capillary XRPD data and validate the extracted lattice parameters used as a reference for the subsequent in situ RH variation experiments. The diffraction signal remained sharp and well defined throughout the capillary measurements, confirming that the polycrystalline precipitates exhibit good crystallinity.

### 3.2. Gradual Relative Humidity Variation Experiments

#### 3.2.1. Evolution of the XRPD Pattern

**Figure 5:**
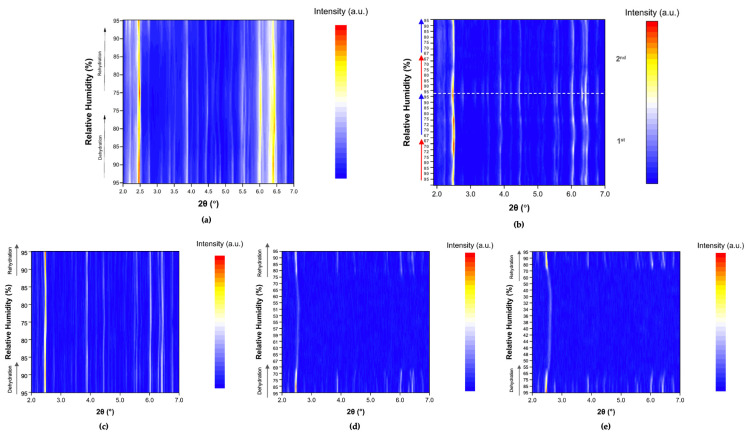
Surface plots of XRPD data collected from tetragonal HEWL polycrystalline samples (space group *P*4_3_2_1_2) during gradual dehydration and rehydration cycles: (**a**) Series 1—Cycle 1, (**b**) Series 2—Cycles 1 and 2, (**c**) Series 5—Cycle 5, (**d**) Series 5—Cycle 6 and (**e**) Series 5—Cycle 7. Diffraction intensity is displayed as a function of 2θ and RH, with the *y*-axis representing successive RH levels applied during each experimental cycle.

The evolution of the diffraction patterns of tetragonal HEWL during gradual dehydration and rehydration cycles is illustrated in the surface plots shown in Figure 5. For all experimental series examined, the overall diffraction motif remains unchanged throughout all the RH variations, indicating preservation of the tetragonal *P*4_3_2_1_2 crystalline symmetry and absence of any phase transition.

In Series 1 and Series 2, only subtle peak shifts are observed upon gradual dehydration, predominantly toward lower 2θ values as RH decreases. These shifts are smooth and continuous, reflecting a progressive structural response of the lattice to dehydration. On the other hand, upon rehydration, the peak positions return close to their initial locations, indicating a largely reversible behavior of the crystalline framework. At lower RH levels, particularly below approximately 70% RH, a gradual weakening of the diffraction intensities is observed, suggesting partial degradation of crystallinity. This effect becomes more pronounced in Series 5, especially after the sample has already experienced multiple prior non-gradual RH variation cycles. In Cycles 6 and 7, where the RH was reduced to substantially lower values, attenuation of the diffraction signal is evident. However, Bragg reflections remain detectable even at the lowest RH level investigated (30%). Upon rehydration, the diffraction signal progressively recovers, with a marked improvement above 70% RH, indicating partial restoration of crystallinity.

#### 3.2.2. Evolution of Unit Cell Parameters from Pawley Analysis

**Figure 6:**
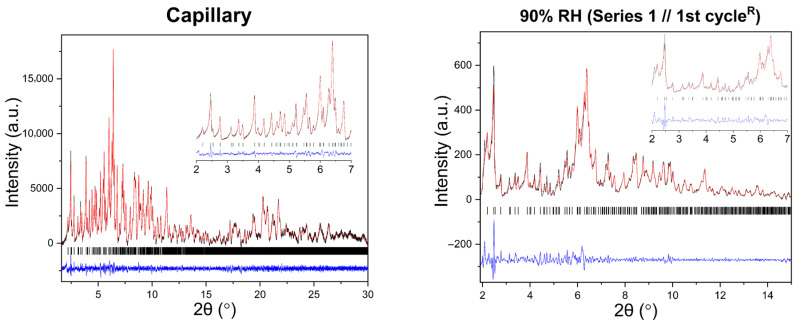

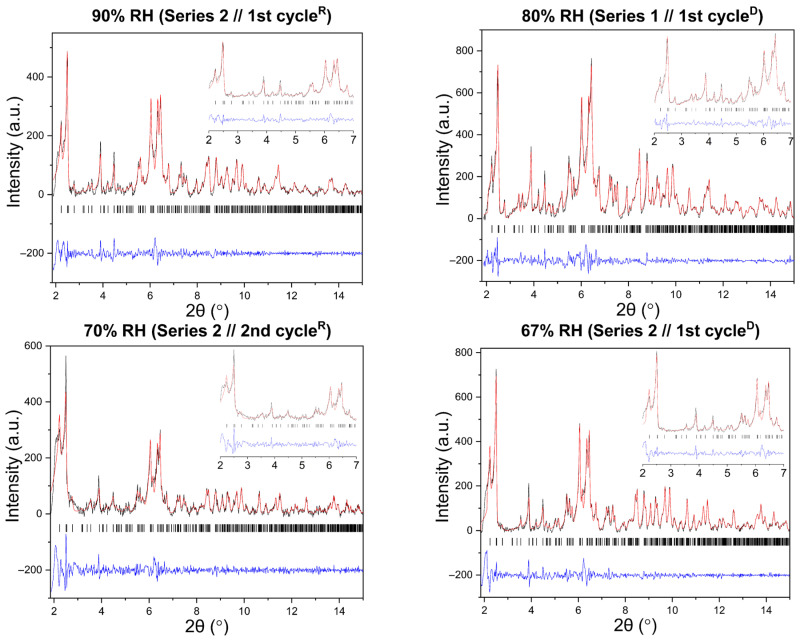
Representative Pawley fits of laboratory XRPD data collected during gradual humidity variation experiments. The black, red and blue lines correspond to the experimental data, the calculated profiles and the difference between them, respectively. Black vertical tic marks indicate the expected Bragg reflections for the tetragonal HEWL polymorph (space group *P*4_3_2_1_2). The main panels display the angular range ~1.8–15° in 2θ, while the insets highlight the low-angle region between 2° and 7° in 2θ. Pawley fit obtained from capillary measurements under ambient conditions is included.

Representative Pawley fits of XRPD patterns collected during gradual humidity variation experiments are shown in Figure 6. In all cases, Pawley analysis yielded satisfactory fits, confirming the persistence of the tetragonal *P*4_3_2_1_2 polymorph (Supplementary Materials, Tables S1, S3, S5, S23, S25 and S27). No additional reflections or peak splitting indicative of phase transitions were detected.

**Figure 7:**
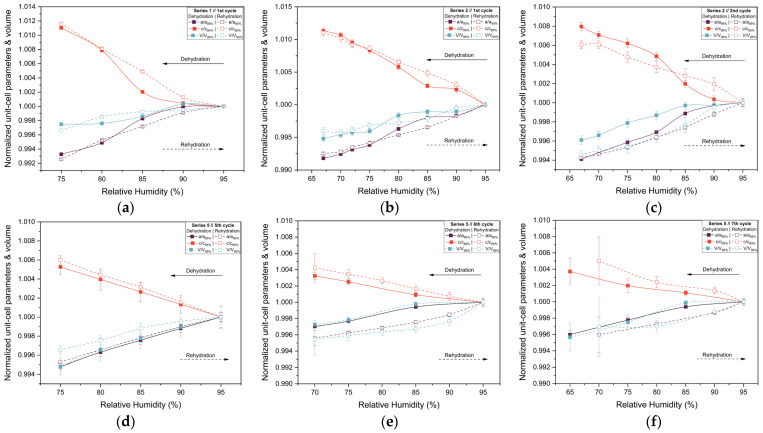
Evolution of the normalized unit cell parameters *a* and *c* and unit cell volume (*V*) of tetragonal HEWL during gradual dehydration and rehydration cycles. Results are shown for (**a**) Series 1—Cycle 1, (**b**) Series 2—Cycle 1, (**c**) Series 2—Cycle 2, (**d**) Series 5—Cycle 5, (**e**) Series 5—Cycle 6 and (**f**) Series 5—Cycle 7. Normalization was performed with respect to unit cell parameters at 95% RH, using the 95% RH value of the dehydration branch for dehydration steps and the 95% RH value of the rehydration branch for rehydration steps within each cycle. This representation allows direct comparison of the evolution of the lattice parameters. The lines and arrows are guides to the eye.

The evolution of normalized unit cell parameters and unit cell volume are summarized in Figure 7. For all series and cycles examined, the lattice parameters exhibit a smooth and continuous response to changes in RH. Specifically, gradual dehydration leads to small but systematic anisotropic variations in the unit cell, with shifts becoming more pronounced as RH decreases below 70%. During rehydration, the lattice parameters largely recover toward their initial values, demonstrating a high degree of reversibility. Nonetheless, minor deviations from original lattice dimensions persist in some cycles, indicating that structural recovery is not always complete, particularly after exposure to lower RH levels or after repeated humidity cycling. The comparison between Series 2 and Series 5 highlights the effect of cumulative experimental history. While the Series 2 sample retained satisfactory diffraction quality and smooth lattice evolution down to 67% RH, the Series 5 sample exhibits earlier weakening of diffraction intensity, consistent with increased mechanical stress from repeated humidity fluctuations. For this reason, Pawley refinement was performed only for diffraction patterns exhibiting clearly identifiable Bragg reflections and sufficient signal-to-noise ratio to ensure reliable profile fitting. At lower RH values (<65%), significant loss of diffraction intensity and peak broadening were observed, preventing reliable refinement.

#### 3.2.3. Percentage Variations and Reproducibility

**Figure 8:**
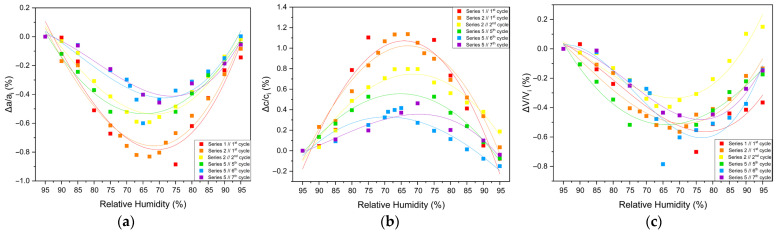
Illustrations of percentage variations in the unit cell parameters and volume of tetragonal HEWL during gradual dehydration–rehydration cycles. The relative changes are expressed as [(Δ*x/x_i_*) × 100%], where *x* corresponds to the unit cell parameters (**a**) *a*, (**b**) *c* and (**c**) unit cell volume (*V*). Additionally, *x_i_* denotes the initial value at 95% at the beginning of the dehydration process for each cycle. Data from all gradual cycles are included (Series 1, 2 and 5). Lines are provided as visual guides.

The structural response of tetragonal HEWL to gradual dehydration and rehydration was quantified through the analysis of percentage variations of unit cell parameters and unit cell volume (Figure 8 and Table 3; Supplementary Materials, Tables S2, S4, S6, S24, S26 and S28). Across all gradual cycles examined (Series 1, 2 and 5), the magnitude of lattice modifications remained limited. The *a*-axis exhibited decreases ranging from approximately −0.40% to −0.82%, while the *c*-axis increased by +0.37% to +1.13%. Correspondingly, the unit cell volume showed modest reductions, varying between −0.25% and −0.79%. These values indicate a pronounced anisotropic lattice response, with the *c*-axis displaying the largest relative changes.

The extent of lattice modification increased systematically with decreasing RH. Cycles reaching lower minimum humidity levels (Series 2—Cycles 1 and 2 and Series 5—Cycles 6 and 7) exhibited larger absolute variations compared to cycles limited to higher RH ranges (95% → 75%). In particular, Series 2—Cycle 1 showed the largest expansion of the *c*-axis (+1.13%), accompanied by reductions in the *a*-axis (−0.82%) and volume (−0.52%). For Series 5—Cycles 6 and 7, which extended to lower RH values, reliable Pawley refinements could only be performed down to 65% RH, due to progressive degradation of diffraction quality at lower humidity levels. Nevertheless, within this accessible range, the lattice variations followed the same anisotropic trends observed in other gradual cycles, with *a* decreasing by up to −0.60% and *c* increasing by up to +0.42%.

Upon rehydration, the lattice parameters exhibited a substantial recovery toward their initial values, indicating that the structural changes induced by dehydration are largely reversible. However, small residual deviations persisted in some cycles, suggesting mild hysteresis effects and possible cumulative structural adjustments. Importantly, all observed lattice modifications evolved smoothly with RH, without abrupt discontinuities, while the limited magnitude of percentage variations and their reproducibility across cycles confirm the high structural resilience of tetragonal HEWL under gradual environmental humidity changes.

### 3.3. Non-Gradual Relative Humidity Variation Experiments

#### 3.3.1. Evolution of the XRPD Patterns

**Figure 9:**
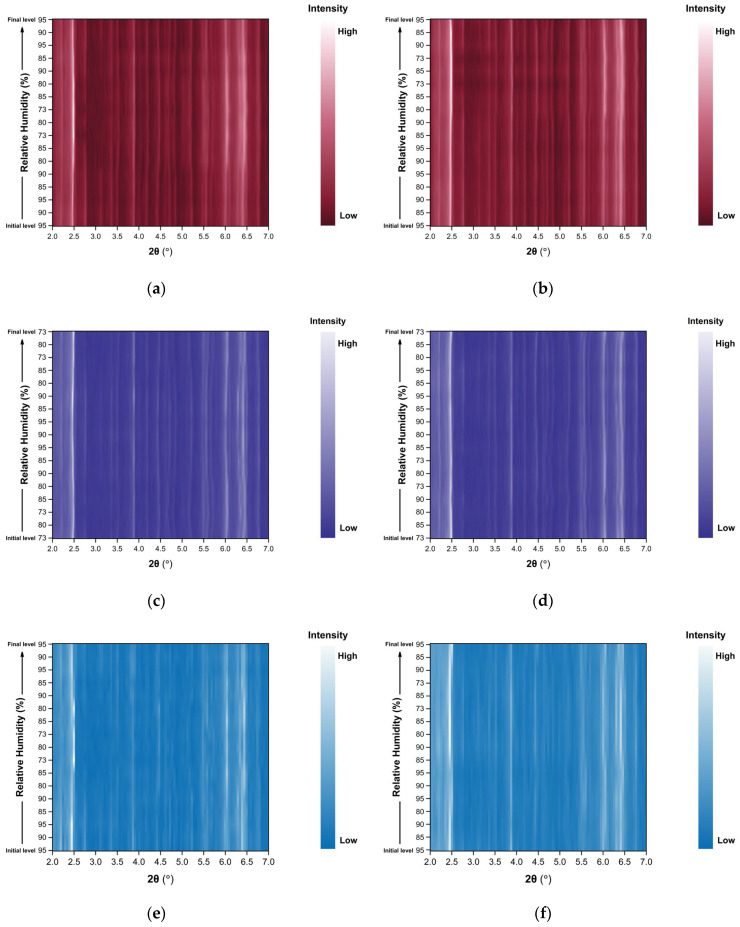

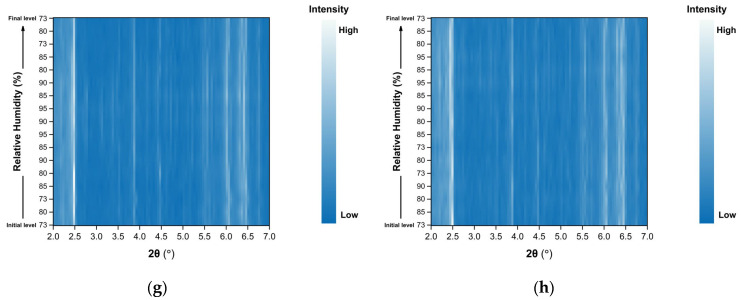
Surface plots of XRPD data collected from tetragonal HEWL polycrystalline samples (space group *P*4_3_2_1_2) during non-gradual cycles: (**a**) Series 3—Cycle 1, (**b**) Series 3—Cycle 2, (**c**) Series 4—Cycle 1, (**d**) Series 4—Cycle 2, (**e**) Series 5—Cycle 1, (**f**) Series 5—Cycle 2, (**g**) Series 5—Cycle 3 and (**h**) Series 5—Cycle 4. Diffraction intensity is displayed as a function of 2θ and RH, with the *y*-axis representing the successive RH levels applied during each experimental cycle.

Surface plots of the XRPD data collected during the non-gradual RH variation experiments for Series 3, 4 and 5 are shown in Figure 9. Small but also systematic shifts in diffraction peak positions are observed upon RH changes, while the overall diffraction pattern remains unchanged throughout all cycles. No emergence or disappearance of Bragg reflections is detected, indicating the absence of phase transitions and the preservation of the tetragonal crystal symmetry (space group *P*4_3_2_1_2) during the entire course of the experiments.

Importantly, no significant degradation of diffraction intensity is observed, and the samples retain their crystallinity throughout all non-gradual RH variation cycles, even when subjected to relatively large humidity steps (for instance 90%→73%).

#### 3.3.2. Evolution of Unit Cell Parameters from Pawley Analysis

**Figure 10:**
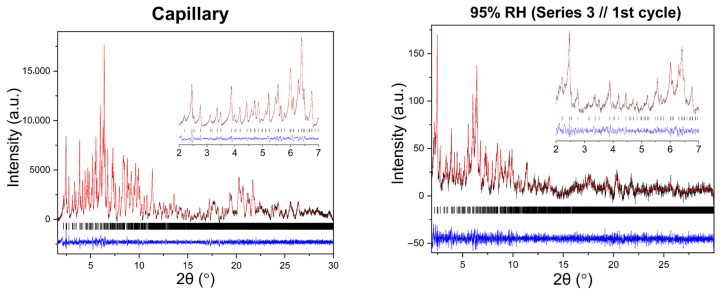

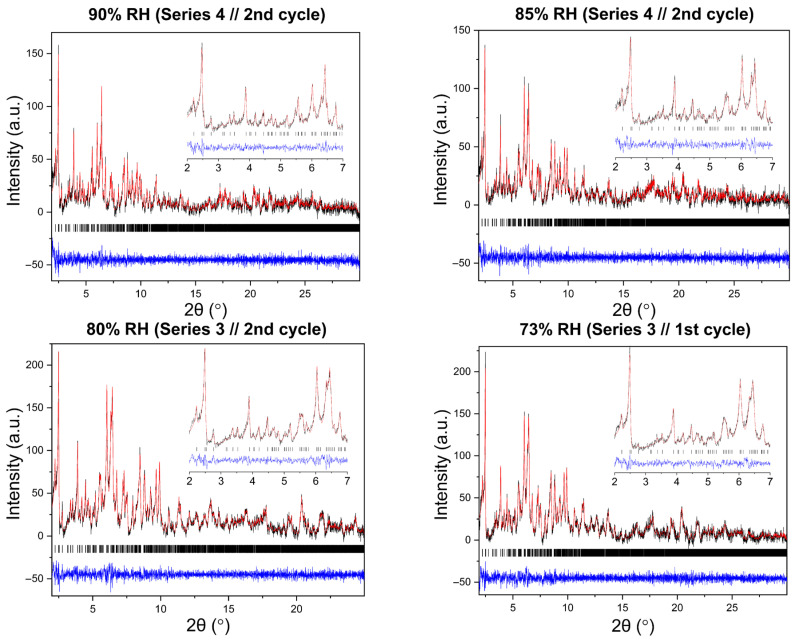
Representative Pawley fits of laboratory XRPD data collected during non-gradual humidity variation experiments. The black, red and blue lines correspond to the experimental data, the calculated profiles and the difference between them, respectively. Black vertical tic marks indicate the expected Bragg reflections for the tetragonal HEWL polymorph (space group *P*4_3_2_1_2). The main panels display the angular range ~1.8–30° in 2θ, while the insets highlight the low-angle region between 2° and 7° in 2θ. Pawley fit obtained from capillary measurements under ambient conditions is included.

Pawley analysis of all XRPD datasets further confirms the persistence of the tetragonal HEWL polymorph (Supplementary Materials, Tables S7, S9, S11, S13, S15, S17, S19 and S21). Representative Pawley fits obtained at selected RH levels are shown in Figure 10, demonstrating satisfactory agreement between experimental and calculated profiles for all series and cycles.

**Figure 11:**
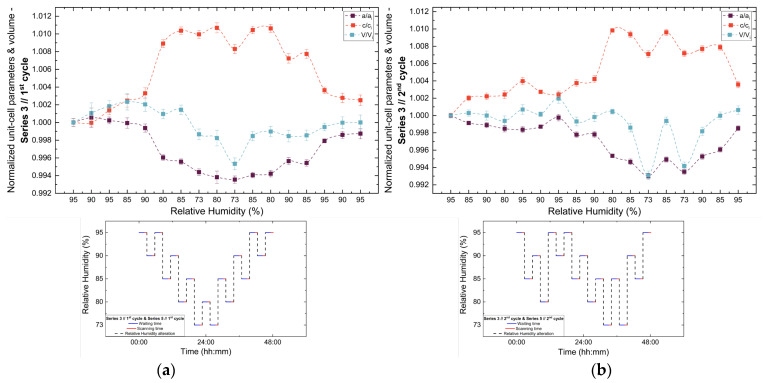
Evolution of normalized unit cell parameters (*a/a_i_*, *c/c_i_*) and unit cell volume (*V/V_i_*) of tetragonal HEWL during the (**a**) 1st and (**b**) 2nd non-gradual RH variation cycles of Series 3. For each cycle, the normalization was performed with respect to the value of each unit cell parameter at the initial RH level of that cycle. The RH protocol applied in each cycle is indicated within each panel to facilitate correlation between lattice response and humidity changes. The lines are guides to the eye.

**Figure 12:**
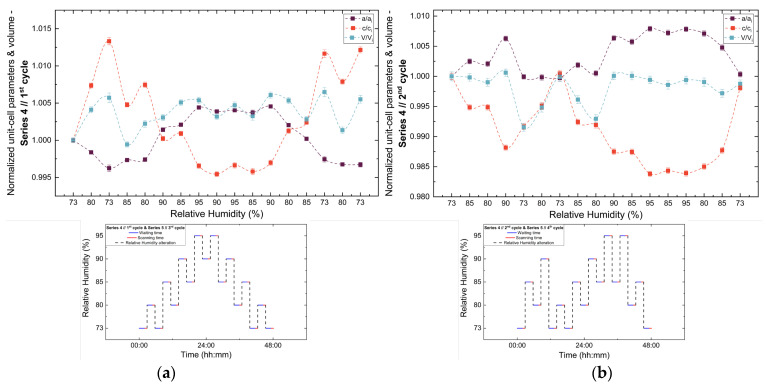
Evolution of the normalized unit cell parameters (*a/a_i_*, *c/c_i_*) and unit cell volume (*V/V_i_*) of tetragonal HEWL during the (**a**) 1st and (**b**) 2nd non-gradual RH variation cycles of Series 4. For each cycle, the normalization was performed with respect to the value of each unit cell parameter at the initial RH level of that cycle. The RH protocol applied in each cycle is indicated within each panel to facilitate correlation between lattice response and humidity changes. The lines are guides to the eye.

**Figure 13:**
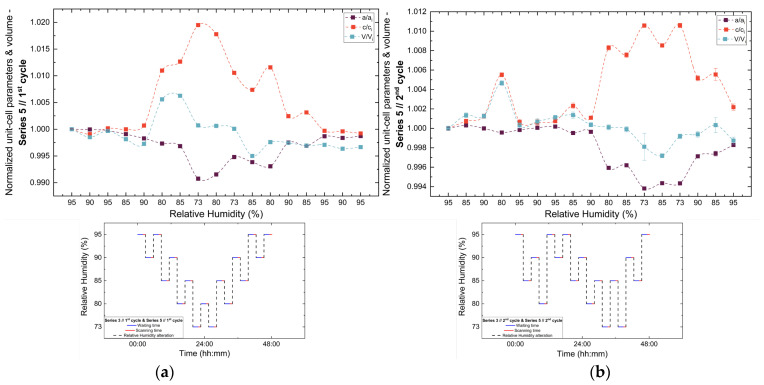

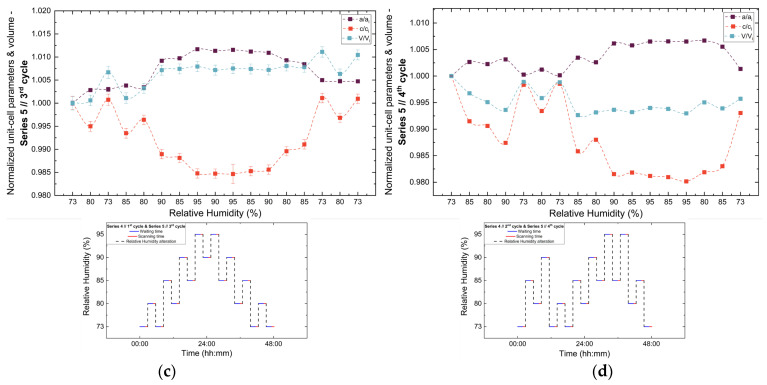
Evolution of the normalized unit cell parameters (*a/a_i_*, *c/c_i_*) and unit cell volume (*V/V_i_*) of tetragonal HEWL during the (**a**) 1st, (**b**) 2nd, (**c**) 3rd and (**d**) 4th non-gradual RH variation cycles of Series 5. For each cycle, the normalization was performed with respect to the value of each unit-cell parameter at the initial RH level of that cycle. The RH protocol applied in each cycle is indicated within each panel to facilitate correlation between lattice response and humidity changes. The lines are guides to the eye.

Analysis of the refined lattice parameters reveals that RH variations induce measurable changes in unit cell dimensions. As illustrated in Figure 11, Figure 12 and Figure 13, the *a* (=*b*) and c lattice parameters respond anisotropically to RH changes. Specifically, decreasing RH leads to a reduction in the *a*-axis and a concomitant expansion of the *c*-axis, whereas the reverse behavior is observed upon rehydration. This anisotropic lattice response is consistently reproduced across Series 3, 4 and 5.

#### 3.3.3. Percentage Variations and Reproducibility

**Figure 14:**
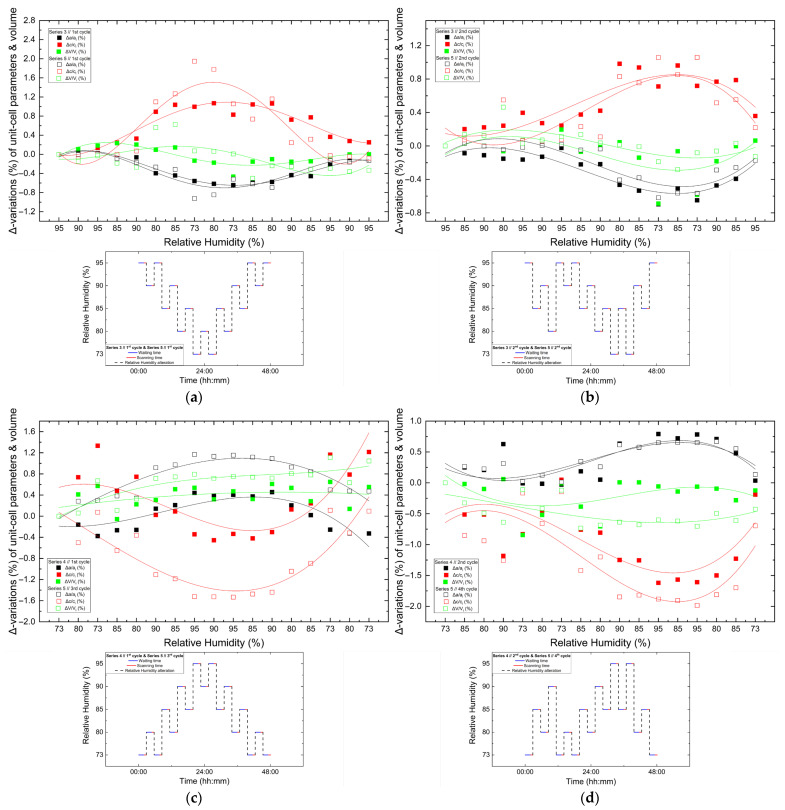
Comparison of percentage variations [(Δ*x/x_i_*) × 100%] of unit cell parameters and unit cell volume of tetragonal HEWL during non-gradual RH variation cycles collected using identical experimental protocols. Panels show: (**a**) Series 3—Cycle 1 and Series 5—Cycle 1, (**b**) Series 3—Cycle 2 and Series 5—Cycle 2, (**c**) Series 4—Cycle 1 and Series 5—Cycle 3 and (**d**) Series 4—Cycle 2 and Series 5—Cycle 4. For each cycle, *x_i_* corresponds to the value of the respective unit cell parameter at the initial RH level of the cycle. All unit cell parameters and volumes (*a*, *c* and *V*) are shown within each panel to enable direct comparison under equivalent humidity pathways. The RH variation protocol applied in each case is included within each panel. Trend lines are provided as visual guides.

The percentage variations of the unit cell parameters (Supplementary Materials, Tables S8, S10, S12, S14, S16, S18, S20 and S22) are summarized in Figure 14. The relative changes remain limited in magnitude, typically within ±2%, highlighting the pronounced structural stability of tetragonal HEWL. Among the lattice parameters, the *c*-axis exhibits the largest relative variations in all cycles, indicating an enhanced structural sensitivity along this crystallographic direction. In contrast, changes in unit cell volume appear to be primarily governed by variations in the *a*-axis, resulting in smoother evolution without abrupt discontinuities.

Comparison between cycles performed under identical RH variation protocols reveals a high degree of reproducibility. While the overall trends of lattice evolution remain consistent, minor deviations and small hysteresis effects are observed in certain cases, suggesting that the structural response of HEWL is largely reversible but may include subtle adjustments related to the hydration–dehydration history of the sample. This behavior is particularly evident when comparing the 3rd cycle of Series 5 with the 1st cycle of Series 4, which followed identical RH pathways, but were performed on samples with different prior exposure histories.

## 4. Discussion

In the present study, an in situ XRPD study was performed to investigate the structural response of tetragonal HEWL under controlled variation in RH using a laboratory X-ray diffraction system. Polycrystalline samples of tetragonal HEWL were subjected to a series of gradual dehydration–rehydration and non-gradual protocols, including repeated RH variations, in order to assess lattice evolution, reproducibility and tolerance to environmental changes. Analysis of the XRPD data provided quantitative information on the variation of unit cell parameters and volume as a function of RH.

Across all experimental protocols explored, the tetragonal HEWL polymorph (space group *P*4_3_2_1_2) retained its symmetry with no evidence of phase transitions throughout the investigated RH range. Variations in RH resulted in continuous modifications of the unit cell parameters, indicating that dehydration primarily affects intermolecular spacing and packing rather than inducing abrupt structural rearrangements. These observations are consistent with previous crystallographic studies on the lysozyme, which demonstrated that dehydration initially leads to lattice parameter changes driven by solvent distribution, while the overall protein fold remains largely preserved [8,44,65].

**Figure 15:**
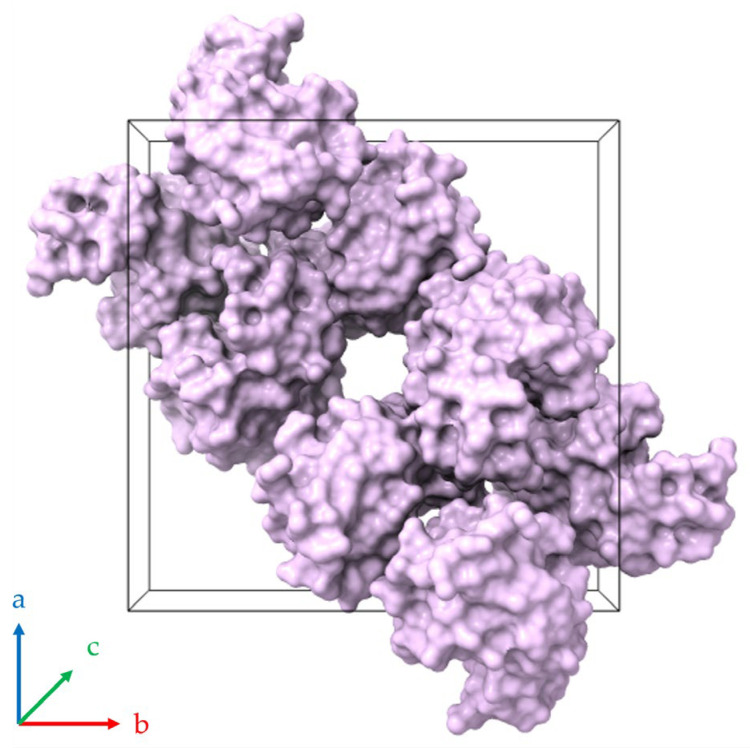
Molecular packing of HEWL within the tetragonal unit cell (space group *P*4_3_2_1_2; PDB ID 1jis; [39,55]). The black three-dimensional box defines the crystallographic unit cell, which contains eight HEWL molecules depicted as surface representations colored lilac. A continuous (linear) solvent channel extends through the crystal lattice parallel to the *c*-axis (perpendicular to the page). Axes *a*, *b* and *c* are indicated by blue, red and green respectively.

A pronounced anisotropic lattice response was observed in all series of experiments. Upon dehydration, the *a* (=*b*)-axis lattice parameter consistently decreased, whereas the *c*-axis increased, with the opposite trend occurring during rehydration. Similar anisotropic behavior has been reported previously for HEWL under varying environmental conditions, including changes in pH, cryoprotectant concentration and RH [38,39,42,66]. This response can be attributed to the crystallographic arrangement of solvent channels and intermolecular contacts within the tetragonal lattice, where solvent redistribution affects specific crystallographic directions to a greater extent [39] (Figure 15). Extended solvent channels run parallel to the crystallographic *c*-axis, while intermolecular contacts within the *ab* plane are comparatively more closely packed. Consequently, the hydration-induced changes in solvent organization are expected to affect the channel direction differently from the more densely packed lateral contacts, leading to contraction in the *a* (=*b*) direction and concomitant expansion along the *c*-axis.

The role of hydration water is central to the observed structural behavior. Structural studies on the lysozyme have shown that bulk solvent located within solvent channels is preferentially removed upon dehydration, whereas a subset of tightly bound water molecules remains associated with specific regions of the protein surface. These water molecules participate in invariant hydration sites and water-mediated interactions that stabilize both tertiary structure and crystal packing [44,67]. As dehydration proceeds, removal of the bulk solvent allows the lattice to adapt through gradual changes in intermolecular distances. At lower hydration levels, disruption of more strongly bound water molecules may occur, potentially leading to larger structural changes [68]. This hierarchy of solvent loss provides a plausible mechanistic explanation for the smooth lattice evolution observed at moderate RH levels and the degradation of diffraction signal quality at lower RH during gradual dehydration.

The magnitude of the observed lattice variations remained limited, typically within ±2% for both unit cell parameters and volume, even at the lowest RH levels accessed. This behavior indicates a high degree of structural stability for the tetragonal HEWL polymorph and agrees with earlier studies showing that significant disruption of crystal order occurs only after extensive depletion of hydration water critical for maintaining crystal contacts [65,69]. Single-crystal investigations have further demonstrated that dehydration leads to increased crystal contacts and remodeling of exposed side chains, while preserving the overall protein fold. Although the present XRPD data do not allow structural interpretation at atomic resolution, the observed lattice responses are consistent with such solvent-mediated adaptation mechanisms.

The behavior of tetragonal HEWL observed differs from that reported for other lysozyme polymorphs, particularly monoclinic forms, which exhibit reduced tolerance to dehydration. In monoclinic HEWL crystals, gradual dehydration up to 75% RH has been shown to result in irreversible loss of crystallinity and collapse of the crystal lattice [38]. In contrast, tetragonal HEWL retained crystalline order over a broader RH range. This difference can be attributed to polymorph-specific hydration architecture and solvent channel organization, which influence the capacity of the lattice to respond to solvent redistribution during dehydration.

A central aspect of the present study is the systematic comparison between gradual and non-gradual RH variation protocols. During gradual dehydration and rehydration cycles, the tetragonal HEWL lattice exhibited smooth and highly reproducible evolution of unit cell parameters. Diffraction patterns remained well defined down to 67% RH, while more pronounced peak shifts and degradation of crystallinity were observed below 65% RH during later gradual cycles in Series 5. These changes were not fully reversible upon rehydration, indicating that extended or repeated exposure to low RH can lead to loss of crystalline order. Similar trends have been reported in previous XRPD studies of HEWL, where gradual dehydration preserved crystallinity over wider RH ranges compared with rapid RH changes, provided that sufficient equilibration time was allowed [38,39].

Non-gradual RH variation protocols, involving larger humidity steps, probed the response of the lattice under more rapid environmental changes. Crystallinity was preserved throughout all non-gradual cycles examined, but history-dependent effects became evident in subsequent measurements. Specifically, during the later gradual cycles of Series 5 (Cycles 6 and 7), which were performed after four preceding non-gradual cycles and one gradual cycle, the diffraction signal degraded at low RH levels (below 65%), limiting the Pawley analysis. This indicates that loss of crystallinity in our experiments is associated with a combination of hydration history and access to low RH levels, rather than being determined solely by the rate of RH variation. A related study of our team on tetragonal polycrystalline HEWL [39] similarly emphasizes that the apparent reversibility and data quality depend on both the RH range explored and the time required for equilibration at each RH level.

The presence of hysteresis between dehydration and rehydration branches provides additional evidence for history-dependent structural effects. Although rehydration largely restores the initial lattice dimensions, lattice evolution during rehydration does not always retrace the dehydration path, especially after multiple cycles. This behavior implies that rearrangements of solvent networks and crystal contacts induced by humidity are path-dependent to some extent. Repeated dehydration and rehydration may progressively reorganize solvent distributions within the lattice, leading to a stable yet subtly modified structural state. Comparable observations have been reported in other protein polycrystalline systems subjected to environmental changes, including insulin complexes and pharmaceutical peptides [40,41].

Beyond the specific case of HEWL, the present results highlight the broader methodological value of in situ XRPD for investigating the structural responses driven by hydration in protein crystals. Controlled in situ XRPD experiments enable continuous and reproducible exploration of hydration-dependent structural landscapes, allowing identification of transient states and history-dependent effects that are inaccessible to static measurements. From an applied perspective, these capabilities are directly relevant to the handling and formulation of protein-based materials, particularly in pharmaceutical and biotechnological contexts where polycrystalline suspensions and microcrystalline solids are increasingly employed. Because exposure to RH variations during manufacturing, storage and transport is often unavoidable, understanding how such variations influence crystal stability is essential for defining processing and handling conditions.

Given the potential impact of uncontrolled humidity variations on pharmaceutical products, particularly those formulated as crystalline or microcrystalline solids, systematic studies of RH provide valuable insight into product stability and structural robustness. Changes in hydration state and lattice organization can influence key physicochemical properties of solid pharmaceutical forms, which in turn may affect parameters relevant to their ADME profiles [70,71], especially absorption, where solid-state properties play a critical role. In this context, in situ RH-dependent diffraction experiments contribute to a mechanistic understanding of how environmental humidity influences crystalline stability, thereby supporting the rational design of formulations and handling protocols that aim to ensure consistent structural properties and predictable in vivo performance.

In addition to humidity variation, temperature is another critical environmental parameter that can significantly influence the structural behavior of protein crystals [40]. Future in situ XRPD studies investigating tetragonal HEWL under controlled temperature variation, either independently or in combination with RH variation, will provide valuable insights into the interplay between thermal effects, hydration dynamics and lattice stability. Further experiments are planned to improve the resolution of XRPD data and to further assess the reproducibility of the observed structural responses. In this context, the substantial enhancement in synchrotron XRPD data quality following the upgrade of the ESRF fourth-generation storage ring, together with the implementation of advanced data-processing strategies at beamline ID22 [72], opens new prospects for more efficient analysis and improved structural sensitivity. High resolution synchrotron measurements under non-ambient conditions are expected to enable more accurate determination of lattice responses and to provide deeper insight into the mechanisms governing structural changes induced by hydration. Such developments will facilitate the optimization of dehydration protocols and contribute to a more comprehensive understanding of protein crystal stability, with potential implications for pharmaceutical research and formulation development.

## 5. Conclusions

The structural response of tetragonal HEWL polycrystalline samples to controlled RH variations was systemically examined using in situ XRPD, encompassing both gradual and non-gradual protocols as well as repeated RH cycling. Across all experimental conditions, the tetragonal polymorph preserved its crystalline symmetry, while RH variation induced anisotropic evolution of the unit cell parameters. The lattice response proved highly reproducible when identical humidity protocols were applied to different samples, indicating that the observed behavior reflects intrinsic properties of the tetragonal HEWL lattice. Although partial degradation of crystallinity was observed following extended exposure to low RH levels and after multiple humidity cycles, the structural response remained largely reversible over a broad RH range. These findings demonstrate that hydration-driven solvent redistribution and reorganization of intermolecular contacts govern the structural adaptation of tetragonal HEWL under non-ambient conditions. The comparative application of gradual and non-gradual RH protocols demonstrates that lattice stability depends not only on the absolute humidity level, but also on the hydration pathway and prior exposure history. Repeated RH cycling resulted in measurable hysteresis between dehydration and rehydration branches indicating history-dependent lattice responses.

More broadly, the results emphasize the methodological value of in situ XRPD as a robust approach for investigating structural behaviors induced by hydration in polycrystalline materials. The ability to monitor lattice evolution continuously under controlled and reversible environmental conditions enables direct assessment of stability, reproducibility and tolerance thresholds that are difficult to capture using static or single-crystal techniques. Such insights are particularly relevant for protein-based materials encountered in pharmaceutical and biotechnological contexts, where exposure to humidity fluctuations is present.

Overall, the findings establish tetragonal HEWL as a structurally resilient protein crystal system and demonstrate that in situ XRPD provides a powerful framework for probing protein and environment interactions under realistic conditions. This approach offers a foundation for future studies aimed at correlating hydration dynamics with structural stability and supports the broader application of diffraction experiments under controlled environment conditions in the characterization of macromolecular crystalline materials.

## Figures and Tables

**Table 1:** Crystallization conditions for HEWL samples.

**Method**	Batch, salting-out
**Container**	Eppendorf tubes
**Initial concentration of HEWL**	200 mg mL^−1^
**Final composition**	100 mg mL^−1^ HEWL 1.5 M sodium chloride 10% *w/v* polyethylene glycol 6000 25 mM sodium acetate
**Final volume**	0.5 mL (0.25 mL protein solution + 0.25 mL precipitation solution)
**Temperature**	RT (~298 K)

**Table 2:** The in situ XRPD data collection parameters for all series of experiments and their respective cycles.

**Series 1**	**Dehydration**	**Rehydration**	**RH% Step**	**Waiting Time**	**Scans/RH Level**
1st Cycle	95%→75%	75%→95%	5%	120 min	20
**Series 2**	**Dehydration**	**Rehydration**	**RH% Step**	**Waiting Time**	**Scans/RH Level**
1st Cycle	95%→67%	67%→95%	5%,3%,2%	60 min	10
2nd Cycle	95%→67%	67%→95%	5%, 3%	60 min	5
**Series 3**	**Dehydration**	**Rehydration**	**RH% Step**	**Waiting Time**	**Scans/RH Level**
1st Cycle	Non-gradual		7%,5%	120 min	5
2nd Cycle	Non-gradual		7%,5%	120 min	5
**Series 4**	**Dehydration**	**Rehydration**	**RH% Step**	**Waiting Time**	**Scans/RH Level**
1st Cycle	Non-gradual		7%,5%	120 min	5
2nd Cycle	Non-gradual		7%,5%	120 min	5
**Series 5**	**Dehydration**	**Rehydration**	**RH% Step**	**Waiting Time**	**Scans/RH Level**
1st Cycle	Non-gradual		7%,5%	120 min	5
2nd Cycle	Non-gradual		7%,5%	120 min	5
3rd Cycle	Non-gradual		7%,5%	120 min	5
4th Cycle	Non-gradual		7%,5%	120 min	5
5th Cycle	95%→75%	75%→95%	5%	120 min	5
6th Cycle	95%→51%	51%→95%	5%,4%,2%,1%	120 min	5
7th Cycle	95%→30%	30%→95%	10%,5%,2%	90 min	5

**Table 3:** Percentage variations in the tetragonal HEWL unit cell parameters and unit cell volume upon gradual dehydration. Percentage variations were calculated as [(*x_f_* − *x_i_*)/*x_i_*] × 100%, where *x_i_* corresponds to the unit cell parameter value at 95% RH and *x_f_* to the value at the lowest RH level at which reliable Pawley refinement was possible for each cycle.

	Ser 1—Cyc 1 (95% → 75%)	Ser 2—Cyc 1 (95% → 67%)	Ser 2—Cyc 2 (95% → 67%)	Ser 5—Cyc 5 (95% → 75%)	Ser 5—Cyc 6 (95% → 65%)	Ser 5—Cyc 7 (95% → 65%)
Δ*a/a_i_ (%)*	−0.67	−0.82	−0.59	−0.52	−0.60	−0.40
Δ*c/c_i_* (%)	1.10	1.13	0.80	0.53	0.42	0.37
Δ*V/V_i_* (%)	−0.25	−0.52	−0.39	−0.52	−0.79	−0.43

## Data Availability

The original contributions presented in the study are included in the article/Supplementary Materials; further inquiries can be directed to the corresponding author.

## Supplementary Materials

The following supporting information can be downloaded at: https://www.mdpi.com/article/10.3390/biom16030442/s1, Table S1–S29: The supplementary material contains detailed tables of refined unit-cell parameters (a, c) and unit-cell volume of tetragonal HEWL obtained via Pawley analysis of XRPD data collected at various RH levels. In addition, the percentage variations of the unit-cell parameters and volume are provided.
