# Supplementary material for: In Situ XRPD Investigation of Relative Humidity-Induced Lattice Responses in Tetragonal Hen Egg-White Lysozyme
Source: Biomolecules. 2026 Mar 15;16(3):442. doi: 10.3390/biom16030442 (PMC13023528; doi:10.3390/biom16030442)
Supplement: Supplementary file 1 [file biomolecules-16-00442-s001.zip › biomolecules-4166366-supplementary.pdf]

Table S1. Refined unit-cell parameters and volume of tetragonal HEWL (space group $P4_32_12$) **for the 1st cycle of the 1st series**, as obtained via Pawley analysis. Data were collected using a laboratory X-ray powder diffractometer X'Pert Pro (Malvern Panalytical) equipped with an MHC-trans humidity and temperature chamber (Anton Paar), at a fixed temperature of 294.15 K [λ = 1.540585(3) Å].

Level No.	Relative Humidity (%)	Space Group	Unit-cell parameters				R_{wp} (%)	χ^2
			$a = b$ (Å)	c (Å)	$\alpha = \beta = \gamma$ (°)	V (×10^5 Å^3)		
1	95	$P4_32_12$	79.316(7)	38.399(4)	90	2.4157(3)	2.6811	4.10036
2	90	$P4_32_12$	79.311(4)	38.416(4)	90	2.4165(2)	2.9038	3.58015
3	85	$P4_32_12$	79.180(3)	38.477(4)	90	2.4123(3)	2.5653	3.14556
4	80	$P4_32_12$	78.912(3)	38.701(4)	90	2.4099(3)	2.7318	3.40868
5	75	$P4_32_12$	78.783(4)	38.823(6)	90	2.4096(4)	2.8354	3.56294
6	75	$P4_32_12$	78.614(5)	38.814(6)	90	2.3987(4)	2.5584	3.63856
7	80	$P4_32_12$	78.824(5)	38.680(5)	90	2.4033(4)	2.4938	3.51557
8	85	$P4_32_12$	78.979(4)	38.557(5)	90	2.4051(3)	2.5222	3.44913
9	90	$P4_32_12$	79.131(6)	38.418(7)	90	2.4057(5)	2.3246	3.10779
10	95	$P4_32_12$	79.202(5)	38.369(6)	90	2.4069(4)	2.6468	3.59992

Table S2. Percentage variations of the unit-cell parameters and volume of tetragonal HEWL **for the 1st cycle of the 1st series**. Relative changes are expressed as [$(x_f\text{-}x_i)/x_i$ × 100%], where x_f corresponds to the values of unit-cell parameters (a, c) and volume (V) at a certain RH level and x_i corresponds to the initial values measured at the beginning of the cycle.

Level No.	Relative Humidity (%)	$\Delta a/a_i$ (%)	$\Delta c/c_i$ (%)	$\Delta V/V_i$ (%)
1	95	0.00000%	0.00000%	0.00000%
2	90	-0.00620%	0.04430%	0.03187%
3	85	-0.17120%	0.20321%	-0.13959%
4	80	-0.51045%	0.78716%	-0.23919%
5	75	-0.67235%	1.10407%	-0.25090%
6	75	-0.88557%	1.08071%	-0.70166%
7	80	-0.61976%	0.73278%	-0.51195%
8	85	-0.42496%	0.41173%	-0.43987%
9	90	-0.23270%	0.05031%	-0.41479%
10	95	-0.14393%	-0.07802%	-0.36544%

Table S3. Refined unit-cell parameters and volume of tetragonal HEWL (space group $P4_32_12$) **for the 1st cycle of the 2nd series**, as obtained via Pawley analysis. Data were collected using a laboratory X-ray powder diffractometer X'Pert Pro (Malvern Panalytical) equipped with an MHC-trans humidity and temperature chamber (Anton Paar), at a fixed temperature of 294.15 K [λ = 1.540585(3) Å].

Level No.	Relative Humidity (%)	Space Group	Unit-cell parameters				R_{wp} (%)	χ^2
			$a = b$ (Å)	c (Å)	$\alpha = \beta = \gamma$ (°)	V (×10^5 Å^3)		
1	95	$P4_32_12$	79.005(7)	38.315(9)	90	2.3915(6)	2.3708	3.34094
2	90	$P4_32_12$	78.871(7)	38.40(2)	90	2.389(1)	2.3159	3.04021
3	85	$P4_32_12$	78.848(4)	38.426(6)	90	2.3889(4)	2.2755	3.49812
4	80	$P4_32_12$	78.712(6)	38.54(1)	90	2.3876(8)	2.2150	3.00056
5	75	$P4_32_12$	78.519(6)	38.63(1)	90	2.3818(9)	2.3194	3.07853
6	72	$P4_32_12$	78.462(7)	38.68(1)	90	2.3813(9)	2.1488	3.00246
7	70	$P4_32_12$	78.407(6)	38.72(1)	90	2.3806(7)	2.2779	2.82820
8	67	$P4_32_12$	78.357(6)	38.75(1)	90	2.3791(9)	2.3660	3.67287
9	67	$P4_32_12$	78.348(9)	38.75(2)	90	2.379(1)	1.7951	2.98301
10	70	$P4_32_12$	78.37(2)	38.72(2)	90	2.378(1)	1.8161	2.95428
11	72	$P4_32_12$	78.42(2)	38.68(1)	90	2.379(1)	1.8785	2.97503
12	75	$P4_32_12$	78.48(2)	38.66(1)	90	2.381(1)	2.1950	3.36201
13	80	$P4_32_12$	78.572(7)	38.58(1)	90	2.3818(9)	2.7911	3.61154
14	85	$P4_32_12$	78.666(8)	38.51(2)	90	2.383(1)	2.4440	3.35033
15	90	$P4_32_12$	78.799(7)	38.44(2)	90	2.387(1)	2.2248	3.10327
16	95	$P4_32_12$	78.939(5)	38.328(9)	90	2.3883(6)	2.3823	3.36950

Table S4. Percentage variations of the unit-cell parameters and volume of tetragonal HEWL **for the 1st cycle of the 2nd series**. Relative changes are expressed as [$(x_f-x_i)/x_i \times 100\%$], where x_f corresponds to the values of unit-cell parameters (a, c) and volume (V) at a certain RH level and x_i corresponds to the initial values measured at the beginning of the cycle.

Level No.	Relative Humidity (%)	$\Delta a/a_i$ (%)	$\Delta c/c_i$ (%)	$\Delta V/V_i$ (%)
1	95	0.00000%	0.00000%	0.00000%
2	90	-0.16933%	0.23229%	-0.10688%
3	85	-0.19832%	0.28991%	-0.10751%
4	80	-0.37067%	0.57954%	-0.16471%
5	75	-0.61509%	0.83208%	-0.40451%
6	72	-0.68635%	0.95477%	-0.42626%
7	70	-0.75693%	1.06669%	-0.45754%
8	67	-0.82012%	1.13309%	-0.51896%
9	67	-0.83076%	1.13630%	-0.53728%
10	70	-0.80321%	1.05241%	-0.56441%
11	72	-0.73459%	0.95052%	-0.52720%
12	75	-0.66837%	0.89634%	-0.44783%
13	80	-0.54798%	0.69046%	-0.41003%
14	85	-0.42849%	0.51821%	-0.34117%
15	90	-0.26012%	0.33702%	-0.18428%
16	95	-0.08364%	0.03369%	-0.13356%

Table S5. Refined unit-cell parameters and volume of tetragonal HEWL (space group $P4_32_12$) **for the 2nd cycle of the 2nd series**, as obtained via Pawley analysis. Data were collected using a laboratory X-ray powder diffractometer X'Pert Pro (Malvern Panalytical) equipped with an MHC-trans humidity and temperature chamber (Anton Paar), at a fixed temperature of 294.15 K [λ = 1.540585(3) Å].

**Level No.**	**Relative Humidity (%)**	**Space Group**	**Unit-cell parameters**				**R_{wp} (%)**	**χ^2**
			**$a = b$ (Å)**	**c (Å)**	**$\alpha = \beta = \gamma$ (°)**	**V (×10^5 Å^3)**		
1	95	$P4_32_12$	78.920(6)	38.295(9)	90	2.3851(6)	2.9307	3.01392
2	90	$P4_32_12$	78.895(6)	38.31(1)	90	2.3845(6)	2.6990	2.92179
3	85	$P4_32_12$	78.831(8)	38.37(1)	90	2.3845(7)	2.8614	2.79924
4	80	$P4_32_12$	78.677(9)	38.48(1)	90	2.3820(8)	2.8430	2.71439
5	75	$P4_32_12$	78.59(1)	38.53(2)	90	2.380(1)	2.4748	2.23291
6	70	$P4_32_12$	78.508(9)	38.57(1)	90	2.3770(7)	2.5249	2.37471
7	67	$P4_32_12$	78.45(1)	38.60(1)	90	2.3758(9)	2.4456	2.43462
8	67	$P4_32_12$	78.452(9)	38.60(1)	90	2.3757(7)	2.5148	2.61336
9	70	$P4_32_12$	78.480(9)	38.60(1)	90	2.3768(7)	2.7113	2.79964
10	75	$P4_32_12$	78.54(1)	38.55(2)	90	2.378(2)	2.7016	2.82793
11	80	$P4_32_12$	78.62(1)	38.51(2)	90	2.380(1)	2.8167	2.94764
12	85	$P4_32_12$	78.70(1)	38.47(3)	90	2.383(2)	2.9768	3.05847
13	90	$P4_32_12$	78.81(1)	38.44(2)	90	2.388(2)	2.7832	2.68239
14	95	$P4_32_12$	78.905(8)	38.37(1)	90	2.389(7)	2.5308	2.52326

Table S6. Percentage variations of the unit-cell parameters and volume of tetragonal HEWL **for the 2nd cycle of the 2nd series**. Relative changes are expressed as [$(x_f-x_i)/x_i$ × 100%], where x_f corresponds to the values of unit-cell parameters (a, c) and volume (V) at a certain RH level and x_i corresponds to the initial values measured at the beginning of the cycle.

**Level No.**	**Relative Humidity (%)**	**$\Delta a/a_i$ (%)**	**$\Delta c/c_i$ (%)**	**$\Delta V/V_i$ (%)**
1	95	0.00000%	0.00000%	0.00000%
2	90	-0.03087%	0.03541%	-0.02633%
3	85	-0.11262%	0.19720%	-0.02834%
4	80	-0.30743%	0.48633%	-0.13060%
5	75	-0.41386%	0.62010%	-0.21106%
6	70	-0.52135%	0.70761%	-0.33977%
7	67	-0.58936%	0.79522%	-0.38937%
8	67	-0.59229%	0.79668%	-0.39381%
9	70	-0.55648%	0.79563%	-0.34866%
10	75	-0.48395%	0.66452%	-0.30745%
11	80	-0.38217%	0.56111%	-0.20607%
12	85	-0.27494%	0.46933%	-0.08239%
13	90	-0.13810%	0.37942%	0.10234%
14	95	-0.01860%	0.18718%	0.14989%

Table S7. Refined unit-cell parameters and volume of tetragonal HEWL (space group $P4_32_12$) **for the 1st cycle of the 3rd series**, as obtained via Pawley analysis. Data were collected using a laboratory X-ray powder diffractometer X'Pert Pro (Malvern Panalytical) equipped with an MHC-trans humidity and temperature chamber (Anton Paar), at a fixed temperature of 294.15 K [λ = 1.540585(3) Å].

**Level No.**	**Relative Humidity (%)**	**Space Group**	**Unit-cell parameters**				**R_{wp} (%)**	**χ^2**
			**$a = b$ (Å)**	**c (Å)**	**$\alpha = \beta = \gamma$ (°)**	**V (×10^5 Å^3)**		
1	95	$P4_32_12$	79.15(1)	38.29(1)	90	2.3990(8)	4.6922	1.66648
2	90	$P4_32_12$	79.20(9)	38.290(8)	90	2.402(3)	4.6171	1.48699
3	95	$P4_32_12$	79.17(3)	38.34(2)	90	2.403(1)	4.4226	1.41247
4	85	$P4_32_12$	79.15(4)	38.39(3)	90	2.405(2)	4.8218	1.76040
5	90	$P4_32_12$	79.10(3)	38.42(2)	90	2.404(2)	4.6170	1.65814
6	80	$P4_32_12$	78.84(2)	38.63(1)	90	2.401(1)	4.3674	1.61013
7	85	$P4_32_12$	78.80(2)	38.69(1)	90	2.402(1)	4.1101	1.45628
8	73	$P4_32_12$	78.71(3)	38.67(1)	90	2.396(1)	3.8493	1.42441
9	80	$P4_32_12$	78.66(5)	38.70(2)	90	2.395(2)	4.1070	1.60905
10	73	$P4_32_12$	78.64(3)	38.61(2)	90	2.388(1)	4.1253	1.58010
11	85	$P4_32_12$	78.68(2)	38.69(1)	90	2.395(1)	4.1883	1.52854
12	80	$P4_32_12$	78.70(3)	38.70(1)	90	2.397(1)	4.0910	1.51102
13	90	$P4_32_12$	78.81(3)	38.57(2)	90	2.395(1)	4.2562	1.63380
14	85	$P4_32_12$	78.79(3)	38.59(1)	90	2.396(1)	4.2282	1.61456
15	95	$P4_32_12$	78.99(1)	38.431(8)	90	2.3978(6)	4.2153	1.41810
16	90	$P4_32_12$	79.04(3)	38.40(2)	90	2.399(1)	4.5488	1.58947
17	95	$P4_32_12$	79.05(5)	38.39(2)	90	2.399(2)	4.5037	1.61434

Table S8. Percentage variations of the unit-cell parameters and volume of tetragonal HEWL **for the 1st cycle of the 3rd series**. Relative changes are expressed as [$(x_f - x_i)/x_i \times 100\%$], where x_f corresponds to the values of unit-cell parameters (a, c) and volume (V) at a certain RH level and x_i corresponds to the initial values measured at the beginning of the cycle.

**Level No.**	**Relative Humidity (%)**	**$\Delta a/a_i$ (%)**	**$\Delta c/c_i$ (%)**	**$\Delta V/V_i$ (%)**
1	95	0.00000%	0.00000%	0.00000%
2	90	0.05469%	-0.00282%	0.10663%
3	95	0.02356%	0.13679%	0.18404%
4	85	-0.00639%	0.24666%	0.23385%
5	90	-0.06264%	0.32995%	0.20434%
6	80	-0.39462%	0.89104%	0.09637%
7	85	-0.44300%	1.03656%	0.14339%
8	73	-0.56049%	0.99574%	-0.13318%
9	80	-0.61757%	1.06884%	-0.17562%
10	73	-0.64483%	0.82973%	-0.46640%
11	85	-0.59410%	1.04398%	-0.15302%
12	80	-0.57766%	1.06216%	-0.10204%
13	90	-0.43641%	0.72362%	-0.15356%
14	85	-0.45727%	0.77423%	-0.14523%
15	95	-0.20727%	0.36468%	-0.05094%
16	90	-0.13974%	0.27785%	-0.00217%
17	95	-0.12534%	0.25142%	0.00029%

Table S9. Refined unit-cell parameters and volume of tetragonal HEWL (space group $P4_32_12$) **for the 2nd cycle of the 3rd series**, as obtained via Pawley analysis. Data were collected using a laboratory X-ray powder diffractometer X'Pert Pro (Malvern Panalytical) equipped with an MHC-trans humidity and temperature chamber (Anton Paar), at a fixed temperature of 294.15 K [λ = 1.540585(3) Å].

Level No.	Relative Humidity (%)	Space Group	Unit-cell parameters				R_{wp} (%)	χ^2
			$a = b$ (Å)	c (Å)	$\alpha = \beta = \gamma$ (°)	V (×10[5] Å[3])		
1	95	$P4_32_12$	79.07(1)	38.290(6)	90	2.3941(5)	4.1711	1.42695
2	85	$P4_32_12$	79.00(1)	38.37(1)	90	2.3948(7)	4.1399	1.56918
3	90	$P4_32_12$	78.98(2)	38.38(1)	90	2.394(1)	4.3128	1.65149
4	80	$P4_32_12$	78.95(3)	38.38(2)	90	2.393(1)	4.3582	1.65132
5	95	$P4_32_12$	78.94(3)	38.44(1)	90	2.396(1)	4.2745	1.59539
6	90	$P4_32_12$	78.971(8)	38.395(6)	90	2.3945(4)	4.2047	1.45635
7	95	$P4_32_12$	79.05(3)	38.38(1)	90	2.399(1)	4.5714	1.72516
8	85	$P4_32_12$	78.90(2)	38.43(1)	90	2.392(1)	4.3151	1.67248
9	90	$P4_32_12$	78.90(3)	38.45(1)	90	2.394(1)	4.2726	1.65889
10	80	$P4_32_12$	78.70(1)	38.667(6)	90	2.3952(5)	3.7801	1.13820
11	85	$P4_32_12$	78.65(2)	38.65(1)	90	2.391(1)	4.1294	1.67941
12	73	$P4_32_12$	78.52(2)	38.56(1)	90	2.378(1)	4.0077	1.66624
13	85	$P4_32_12$	78.67(2)	38.66(1)	90	2.393(1)	4.0863	1.67106
14	73	$P4_32_12$	78.56(2)	38.57(1)	90	2.380(1)	4.0332	1.64696
15	90	$P4_32_12$	78.70(2)	38.58(1)	90	2.390(1)	4.3761	1.76322
16	85	$P4_32_12$	78.76(2)	38.59(1)	90	2.3940(9)	4.2848	1.76667
17	95	$P4_32_12$	78.96(2)	38.43(1)	90	2.396(1)	4.4942	1.70760

Table S10. Percentage variations of the unit-cell parameters and volume of tetragonal HEWL **for the 2nd cycle of the 3rd series**. Relative changes are expressed as [(x_f-x_i)/x_i × 100%], where x_f corresponds to the values of unit-cell parameters (a, c) and volume (V) at a certain RH level and x_i corresponds to the initial values measured at the beginning of the cycle.

Level No.	Relative Humidity (%)	$\Delta a/a_i$ (%)	$\Delta c/c_i$ (%)	$\Delta V/V_i$ (%)
1	95	0.00000%	0.00000%	0.00000%
2	85	-0.08667%	0.20219%	0.02861%
3	90	-0.11106%	0.22209%	-0.00042%
4	80	-0.15208%	0.24228%	-0.06236%
5	95	-0.16305%	0.39700%	0.06984%
6	90	-0.12860%	0.27333%	0.01558%
7	95	-0.02389%	0.24411%	0.19619%
8	85	-0.22100%	0.37453%	-0.06863%
9	90	-0.21850%	0.42120%	-0.01717%
10	80	-0.46536%	0.98294%	0.04528%
11	85	-0.53536%	0.93815%	-0.13972%
12	73	-0.69741%	0.71052%	-0.68933%
13	85	-0.50805%	0.96064%	-0.06261%
14	73	-0.64878%	0.71911%	-0.58356%
15	90	-0.47305%	0.76918%	-0.18195%
16	85	-0.39268%	0.78798%	-0.00200%
17	95	-0.14666%	0.35787%	0.06370%

Table S11. Refined unit-cell parameters and volume of tetragonal HEWL (space group $P4_32_12$) **for the 1st cycle of the 4th series**, as obtained via Pawley analysis. Data were collected using a laboratory X-ray powder diffractometer X'Pert Pro (Malvern Panalytical) equipped with an MHC-trans humidity and temperature chamber (Anton Paar), at a fixed temperature of 294.15 K [λ = 1.540585(3) Å].

Level No.	Relative Humidity (%)	Space Group	Unit-cell parameters				R_{wp} (%)	χ^2
			$a = b$ (Å)	c (Å)	$\alpha = \beta = \gamma$ (°)	V (×10⁵ Å³)		
1	73	$P4_32_12$	78.689(8)	38.366(8)	90	2.3756(6)	4.6878	1.64122
2	80	$P4_32_12$	78.56(2)	38.65(1)	90	2.3853(8)	4.2675	1.38859
3	73	$P4_32_12$	78.39(3)	38.88(2)	90	2.389(1)	4.5020	1.48303
4	85	$P4_32_12$	78.48(1)	38.549(8)	90	2.3743(6)	4.2990	1.36077
5	80	$P4_32_12$	78.48(2)	38.65(1)	90	2.3809(9)	4.4777	1.43076
6	90	$P4_32_12$	78.80(2)	38.375(8)	90	2.3829(8)	4.4771	1.40429
7	85	$P4_32_12$	78.85(2)	38.401(8)	90	2.3877(7)	4.5000	1.42332
8	95	$P4_32_12$	79.04(2)	38.23(1)	90	2.3883(8)	4.4595	1.41248
9	90	$P4_32_12$	78.99(1)	38.191(9)	90	2.3831(7)	4.4059	1.39635
10	95	$P4_32_12$	79.01(2)	38.24(1)	90	2.3868(9)	4.8287	1.55227
11	85	$P4_32_12$	78.98(2)	38.20(1)	90	2.383(1)	4.4527	1.45770
12	90	$P4_32_12$	79.05(1)	38.25(1)	90	2.3900(8)	4.9584	1.65050
13	80	$P4_32_12$	78.85(2)	38.41(1)	90	2.3883(8)	4.4019	1.39213
14	85	$P4_32_12$	78.70(1)	38.459(8)	90	2.3823(7)	4.4997	1.40137
15	73	$P4_32_12$	78.49(3)	38.81(2)	90	2.391(1)	4.8288	1.70733
16	80	$P4_32_12$	78.43(2)	38.67(1)	90	2.3788(8)	4.5599	1.42484
17	73	$P4_32_12$	78.43(2)	38.83(1)	90	2.389(1)	4.6479	1.47443

Table S12. Percentage variations of the unit-cell parameters and volume of tetragonal HEWL **for the 1st cycle of the 4th series**. Relative changes are expressed as [$(x_f-x_i)/x_i$ × 100%], where x_f corresponds to the values of unit-cell parameters (a, c) and volume (V) at a certain RH level and x_i corresponds to the initial values measured at the beginning of the cycle.

Level No.	Relative Humidity (%)	$\Delta a/a_i$ (%)	$\Delta c/c_i$ (%)	$\Delta V/V_i$ (%)
1	73	0.00000%	0.00000%	0.00000%
2	80	-0.16198%	0.73680%	0.41072%
3	73	-0.37608%	1.33217%	0.57144%
4	85	-0.26598%	0.47769%	-0.05611%
5	80	-0.25971%	0.74519%	0.22260%
6	90	0.14166%	0.02283%	0.30641%
7	85	0.20909%	0.09029%	0.50926%
8	95	0.44086%	-0.34379%	0.53684%
9	90	0.38744%	-0.45491%	0.31794%
10	95	0.40359%	-0.33582%	0.47028%
11	85	0.37289%	-0.42003%	0.32400%
12	90	0.45534%	-0.30209%	0.60793%
13	80	0.20379%	0.12636%	0.53486%
14	85	0.01944%	0.24318%	0.28216%
15	73	-0.25536%	1.16431%	0.64830%
16	80	-0.32383%	0.78825%	0.13651%
17	73	-0.32870%	1.21522%	0.55094%

Table S13. Refined unit-cell parameters and volume of tetragonal HEWL (space group $P4_32_12$) **for the 2nd cycle of the 4th series**, as obtained via Pawley analysis. Data were collected using a laboratory X-ray powder diffractometer X'Pert Pro (Malvern Panalytical) equipped with an MHC-trans humidity and temperature chamber (Anton Paar), at a fixed temperature of 294.15 K [λ = 1.540585(3) Å].

Level No.	Relative Humidity (%)	Space Group	Unit-cell parameters				R_{wp} (%)	χ^2
			$a = b$ (Å)	c (Å)	$\alpha = \beta = \gamma$ (°)	V (×10^5 Å^3)		
1	73	$P4_32_12$	78.40(3)	38.83(1)	90	2.387(1)	4.2767	1.40871
2	85	$P4_32_12$	78.59(2)	38.634(9)	90	2.3862(7)	4.4699	1.40950
3	80	$P4_32_12$	78.56(2)	38.63(1)	90	2.3843(9)	4.6067	1.43978
4	90	$P4_32_12$	78.89(1)	38.374(7)	90	2.3881(6)	4.4178	1.37932
5	73	$P4_32_12$	78.390(9)	38.51(1)	90	2.3665(9)	4.8518	1.60347
6	80	$P4_32_12$	78.38(3)	38.64(2)	90	2.374(1)	4.5201	1.44645
7	73	$P4_32_12$	78.37(3)	38.85(1)	90	2.386(1)	4.4594	1.44560
8	85	$P4_32_12$	78.54(2)	38.54(1)	90	2.3774(9)	4.3861	1.36060
9	80	$P4_32_12$	78.44(2)	38.52(1)	90	2.370(1)	4.7487	1.47128
10	90	$P4_32_12$	78.89(2)	38.35(1)	90	2.3868(8)	4.6834	1.45573
11	85	$P4_32_12$	78.85(2)	38.35(1)	90	2.3868(9)	4.5319	1.47785
12	95	$P4_32_12$	79.02(2)	38.204(9)	90	2.3852(8)	4.5674	1.39975
13	85	$P4_32_12$	78.96(2)	38.22(1)	90	2.383(1)	4.2413	1.36025
14	95	$P4_32_12$	79.01(2)	38.21(1)	90	2.3852(9)	4.2895	1.31983
15	80	$P4_32_12$	78.95(2)	38.25(1)	90	2.384(1)	4.4472	1.44023
16	85	$P4_32_12$	78.77(2)	38.36(1)	90	2.3799(9)	4.4244	1.38333
17	73	$P4_32_12$	78.42(2)	38.76(1)	90	2.3837(9)	4.2624	1.37929

Table S14. Percentage variations of the unit-cell parameters and volume of tetragonal HEWL **for the 2nd cycle of the 4th series**. Relative changes are expressed as [$(x_f - x_i)/x_i$ × 100%], where x_f corresponds to the values of unit-cell parameters (a, c) and volume (V) at a certain RH level and x_i corresponds to the initial values measured at the beginning of the cycle.

Level No.	Relative Humidity (%)	$\Delta a/a_i$ (%)	$\Delta c/c_i$ (%)	$\Delta V/V_i$ (%)
1	73	0.00000%	0.00000%	0.00000%
2	85	0.24859%	-0.51353%	-0.01831%
3	80	0.20781%	-0.51252%	-0.09863%
4	90	0.62722%	-1.18311%	0.06034%
5	73	-0.00722%	-0.83117%	-0.84550%
6	80	-0.01584%	-0.48737%	-0.51893%
7	73	-0.02839%	0.04952%	-0.00729%
8	85	0.18709%	-0.75773%	-0.38607%
9	80	0.05175%	-0.80758%	-0.70488%
10	90	0.63463%	-1.24942%	0.00792%
11	85	0.57442%	-1.25519%	0.00817%
12	95	0.79019%	-1.61938%	-0.05849%
13	85	0.72116%	-1.56683%	-0.14200%
14	95	0.78260%	-1.60754%	-0.06151%
15	80	0.71004%	-1.49879%	-0.09503%
16	85	0.47841%	-1.22817%	-0.28085%
17	73	0.03464%	-0.19244%	-0.12331%

Table S15. Refined unit-cell parameters and volume of tetragonal HEWL (space group $P4_32_12$) **for the 1st cycle of the 5th series**, as obtained via Pawley analysis. Data were collected using a laboratory X-ray powder diffractometer X'Pert Pro (Malvern Panalytical) equipped with an MHC-trans humidity and temperature chamber (Anton Paar), at a fixed temperature of 294.15 K [λ = 1.540585(3) Å].

Level No.	Relative Humidity (%)	Space Group	Unit-cell parameters				R_{wp} (%)	χ^2
			$a = b$ (Å)	c (Å)	$\alpha = \beta = \gamma$ (°)	V (×10^5 Å^3)		
1	95	$P4_32_12$	79.050(5)	38.207(5)	90	2.3875(3)	5.2100	1.65237
2	90	$P4_32_12$	79.047(9)	38.171(9)	90	2.3839(6)	5.7607	1.79434
3	95	$P4_32_12$	79.031(5)	38.213(5)	90	2.3867(3)	5.3800	1.69273
4	85	$P4_32_12$	78.977(8)	38.21(1)	90	2.3830(7)	5.6653	1.64568
5	90	$P4_32_12$	78.914(8)	38.23(1)	90	2.3810(6)	5.8887	1.80844
6	80	$P4_32_12$	78.838(5)	38.626(7)	90	2.4008(4)	5.9354	1.82989
7	85	$P4_32_12$	78.800(6)	38.690(7)	90	2.4024(5)	5.8072	1.77932
8	73	$P4_32_12$	78.32(1)	38.95(1)	90	2.3892(8)	5.2101	1.61711
9	80	$P4_32_12$	78.38(1)	38.886(9)	90	2.3890(7)	5.1406	1.55543
10	73	$P4_32_12$	78.639(5)	38.610(5)	90	2.3877(3)	6.3902	1.96075
11	85	$P4_32_12$	78.562(7)	38.489(8)	90	2.3755(6)	5.5303	1.70618
12	80	$P4_32_12$	78.501(7)	38.65(1)	90	2.3817(7)	5.3177	1.63453
13	90	$P4_32_12$	78.853(9)	38.30(1)	90	2.3814(8)	5.7111	1.73431
14	85	$P4_32_12$	78.804(8)	38.327(8)	90	2.3802(5)	5.4101	1.78888
15	95	$P4_32_12$	78.946(8)	38.20(1)	90	2.3805(7)	5.8630	1.39663
16	90	$P4_32_12$	78.921(7)	38.191(7)	90	2.3788(5)	6.0257	1.85508
17	95	$P4_32_12$	78.949(9)	38.18(1)	90	2.3795(8)	5.7165	1.67873

Table S16. Percentage variations of the unit-cell parameters and volume of tetragonal HEWL **for the 1st cycle of the 5th series**. Relative changes are expressed as [$(x_f-x_i)/x_i$ × 100%], where x_f corresponds to the values of unit-cell parameters (a, c) and volume (V) at a certain RH level and x_i corresponds to the initial values measured at the beginning of the cycle.

Level No.	Relative Humidity (%)	$\Delta a/a_i$ (%)	$\Delta c/c_i$ (%)	$\Delta V/V_i$ (%)
1	95	0.00000%	0.00000%	0.00000%
2	90	-0.00364%	-0.09336%	-0.14827%
3	95	-0.02409%	0.01631%	-0.03187%
4	85	-0.09207%	-0.00204%	-0.18610%
5	90	-0.17104%	0.06897%	-0.27309%
6	80	-0.26717%	1.09831%	0.55879%
7	85	-0.31604%	1.26525%	0.62618%
8	73	-0.92476%	1.94874%	0.07187%
9	80	-0.84671%	1.77864%	0.06241%
10	73	-0.51988%	1.05563%	0.00762%
11	85	-0.61642%	0.73817%	-0.49994%
12	80	-0.69355%	1.15781%	-0.24050%
13	90	-0.24921%	0.24409%	-0.25491%
14	85	-0.31045%	0.31523%	-0.30668%
15	95	-0.13178%	-0.02879%	-0.29211%
16	90	-0.16249%	-0.03986%	-0.36448%
17	95	-0.12758%	-0.07938%	-0.33420%

Table S17. Refined unit-cell parameters and volume of tetragonal HEWL (space group $P4_32_12$) **for the 2nd cycle of the 5th series**, as obtained via Pawley analysis. Data were collected using a laboratory X-ray powder diffractometer X'Pert Pro (Malvern Panalytical) equipped with an MHC-trans humidity and temperature chamber (Anton Paar), at a fixed temperature of 294.15 K [λ = 1.540585(3) Å].

Level No.	Relative Humidity (%)	Space Group	Unit-cell parameters				R_{wp} (%)	χ^2
			$a = b$ (Å)	c (Å)	$\alpha = \beta = \gamma$ (°)	V (×10⁵ Å³)		
1	95	$P4_32_12$	78.982(6)	38.171(6)	90	2.3811(4)	5.2056	1.55393
2	85	$P4_32_12$	79.006(6)	38.199(6)	90	2.3843(4)	5.0010	1.55107
3	90	$P4_32_12$	78.981(6)	38.219(6)	90	2.3841(4)	5.1745	1.59004
4	80	$P4_32_12$	78.948(6)	38.381(6)	90	2.3922(4)	5.5904	1.66982
5	95	$P4_32_12$	78.969(7)	38.196(9)	90	2.3819(6)	5.9767	2.02704
6	90	$P4_32_12$	78.986(8)	38.19(1)	90	2.3828(7)	4.9614	1.50711
7	95	$P4_32_12$	78.996(5)	38.199(5)	90	2.3838(4)	5.1318	1.55586
8	85	$P4_32_12$	78.944(8)	38.26(1)	90	2.3844(6)	5.0121	1.55039
9	90	$P4_32_12$	78.953(4)	38.212(5)	90	2.3820(3)	5.3971	1.74022
10	80	$P4_32_12$	78.660(7)	38.488(7)	90	2.3814(5)	5.0459	1.50605
11	85	$P4_32_12$	78.682(6)	38.459(8)	90	2.3809(5)	5.4165	1.66672
12	73	$P4_32_12$	78.493(5)	38.575(5)	90	2.377(3)	6.6389	1.98541
13	85	$P4_32_12$	78.536(5)	38.496(4)	90	2.3744(3)	6.0104	1.55976
14	73	$P4_32_12$	78.534(6)	38.575(6)	90	2.3792(4)	5.9351	1.77506
15	90	$P4_32_12$	78.755(9)	38.368(9)	90	2.3797(6)	5.7065	1.73290
16	85	$P4_32_12$	78.78(2)	38.38(2)	90	2.382(2)	8.0095	1.87444
17	95	$P4_32_12$	78.85(1)	38.25(1)	90	2.3781(8)	5.8970	1.75363

Table S18. Percentage variations of the unit-cell parameters and volume of tetragonal HEWL **for the 2nd cycle of the 5th series**. Relative changes are expressed as [$(x_f$-$x_i)/x_i$ × 100%], where x_f corresponds to the values of unit-cell parameters (a, c) and volume (V) at a certain RH level and x_i corresponds to the initial values measured at the beginning of the cycle.

Level No.	Relative Humidity (%)	$\Delta a/a_i$ (%)	$\Delta c/c_i$ (%)	$\Delta V/V_i$ (%)
1	95	0.00000%	0.00000%	0.00000%
2	85	0.03065%	0.07322%	0.13460%
3	90	-0.00081%	0.12701%	0.12540%
4	80	-0.04276%	0.55136%	0.46541%
5	95	-0.01651%	0.06542%	0.03238%
6	90	0.00572%	0.05910%	0.07055%
7	95	0.01835%	0.07511%	0.11184%
8	85	-0.04781%	0.23180%	0.13599%
9	90	-0.03598%	0.10877%	0.03675%
10	80	-0.40675%	0.83011%	0.01155%
11	85	-0.38010%	0.75524%	-0.00924%
12	73	-0.61890%	1.05803%	-0.18899%
13	85	-0.56432%	0.85311%	-0.28192%
14	73	-0.56649%	1.05927%	-0.08244%
15	90	-0.28769%	0.51629%	-0.06119%
16	85	-0.25944%	0.55417%	0.03309%
17	95	-0.17165%	0.21784%	-0.12591%

Table S19. Refined unit-cell parameters and volume of tetragonal HEWL (space group $P4_32_12$) **for the 3rd cycle of the 5th series**, as obtained via Pawley analysis. Data were collected using a laboratory X-ray powder diffractometer X'Pert Pro (Malvern Panalytical) equipped with an MHC-trans humidity and temperature chamber (Anton Paar), at a fixed temperature of 294.15 K [λ = 1.540585(3) Å].

Level No.	Relative Humidity (%)	Space Group	Unit-cell parameters				R_{wp} (%)	χ^2
			$a = b$ (Å)	c (Å)	$\alpha = \beta = \gamma$ (°)	V (×10⁵ Å³)		
1	73	$P4_32_12$	78.06(3)	38.80(4)	90	2.364(3)	4.8211	1.60099
2	80	$P4_32_12$	78.279(7)	38.60(2)	90	2.365(1)	5.2691	1.45279
3	73	$P4_32_12$	78.29(3)	38.82(3)	90	2.380(2)	5.2662	1.72836
4	85	$P4_32_12$	78.357(8)	38.54(1)	90	2.3664(9)	5.3627	1.60534
5	80	$P4_32_12$	78.327(7)	38.655(6)	90	2.3715(4)	5.6511	1.72300
6	90	$P4_32_12$	78.774(5)	38.366(5)	90	2.3808(3)	5.0855	1.56058
7	85	$P4_32_12$	78.817(4)	38.335(4)	90	2.3814(3)	4.7968	1.46461
8	95	$P4_32_12$	78.971(7)	38.204(7)	90	2.3826(5)	5.4233	1.69233
9	90	$P4_32_12$	78.943(7)	38.203(7)	90	2.3808(5)	5.2649	1.63746
10	95	$P4_32_12$	78.960(7)	38.20(7)	90	2.3816(5)	5.2181	1.62378
11	85	$P4_32_12$	78.931(7)	38.223(7)	90	2.3814(5)	4.9814	1.60041
12	90	$P4_32_12$	78.909(7)	38.237(9)	90	2.3809(6)	5.0232	1.59151
13	80	$P4_32_12$	78.784(5)	38.391(4)	90	2.3829(3)	5.0995	1.56899
14	85	$P4_32_12$	78.715(6)	38.449(6)	90	2.3823(4)	4.8752	1.48104
15	73	$P4_32_12$	78.45(1)	38.84(1)	90	2.3901(8)	5.3200	1.65769
16	80	$P4_32_12$	78.430(5)	38.672(4)	90	2.3788(3)	5.1745	1.57018
17	73	$P4_32_12$	78.428(5)	38.832(5)	90	2.3886(3)	5.0534	1.53485

Table S20. Percentage variations of the unit-cell parameters and volume of tetragonal HEWL **for the 3rd cycle of the 5th series**. Relative changes are expressed as [(x_f-x_i)/x_i × 100%], where x_f corresponds to the values of unit-cell parameters (a, c) and volume (V) at a certain RH level and x_i corresponds to the initial values measured at the beginning of the cycle.

Level No.	Relative Humidity (%)	$\Delta a/a_i$ (%)	$\Delta c/c_i$ (%)	$\Delta V/V_i$ (%)
1	73	0.00000%	0.00000%	0.00000%
2	80	0.28248%	-0.50261%	0.06030%
3	73	0.29712%	0.07390%	0.66947%
4	85	0.38171%	-0.65065%	0.10926%
5	80	0.34306%	-0.36061%	0.32421%
6	90	0.91677%	-1.10609%	0.71548%
7	85	0.97174%	-1.18718%	0.74256%
8	95	1.16875%	-1.52282%	0.79253%
9	90	1.13271%	-1.52730%	0.71615%
10	95	1.15414%	-1.53429%	0.75170%
11	85	1.11752%	-1.47407%	0.74032%
12	90	1.08972%	-1.43946%	0.72031%
13	80	0.92921%	-1.04273%	0.80485%
14	85	0.84094%	-0.89362%	0.78025%
15	73	0.49754%	0.11128%	1.10993%
16	80	0.47577%	-0.31785%	0.63292%
17	73	0.47349%	0.09501%	1.04513%

Table S21. Refined unit-cell parameters and volume of tetragonal HEWL (space group $P4_32_12$) **for the 4th cycle of the 5th series**, as obtained via Pawley analysis. Data were collected using a laboratory X-ray powder diffractometer X'Pert Pro (Malvern Panalytical) equipped with an MHC-trans humidity and temperature chamber (Anton Paar), at a fixed temperature of 294.15 K [λ = 1.540585(3) Å].

Level No.	Relative Humidity (%)	Space Group	Unit-cell parameters				R_{wp} (%)	χ^2
			$a = b$ (Å)	c (Å)	$\alpha = \beta = \gamma$ (°)	V (×10⁵ Å³)		
1	73	$P4_32_12$	78.314(4)	39.031(3)	90	2.3938(2)	5.2137	1.58658
2	85	$P4_32_12$	78.522(5)	38.699(4)	90	2.3860(3)	5.2935	1.60179
3	80	$P4_32_12$	78.491(5)	38.665(4)	90	2.3821(3)	5.1455	1.55390
4	90	$P4_32_12$	78.559(5)	38.541(3)	90	2.3786(2)	5.6920	1.71287
5	73	$P4_32_12$	78.335(8)	38.967(7)	90	2.3912(5)	4.7564	1.43389
6	80	$P4_32_12$	78.410(5)	38.775(4)	90	2.3839(3)	5.1743	1.57553
7	73	$P4_32_12$	78.325(4)	38.975(3)	90	2.3910(2)	5.0439	1.52541
8	85	$P4_32_12$	78.585(3)	38.478(2)	90	2.3762(2)	4.9410	1.48799
9	80	$P4_32_12$	78.518(8)	38.56(1)	90	2.3774(7)	5.0556	1.38338
10	90	$P4_32_12$	78.796(4)	38.310(3)	90	2.3786(2)	4.9849	1.48141
11	85	$P4_32_12$	78.766(6)	38.322(5)	90	2.3775(4)	5.1289	1.54976
12	95	$P4_32_12$	78.824(4)	38.297(4)	90	2.3794(3)	5.0266	1.49578
13	85	$P4_32_12$	78.826(8)	38.288(8)	90	2.3790(6)	4.8191	1.46596
14	95	$P4_32_12$	78.824(4)	38.257(3)	90	2.3770(3)	5.5376	1.64104
15	80	$P4_32_12$	78.837(5)	38.324(5)	90	2.3820(3)	4.7815	1.45591
16	85	$P4_32_12$	78.747(5)	38.369(5)	90	2.3792(3)	5.2372	1.56912
17	73	$P4_32_12$	78.420(4)	38.760(4)	90	2.3836(2)	4.9954	1.49505

Table S22. Percentage variations of the unit-cell parameters and volume of tetragonal HEWL **for the 4th cycle of the 5th series**. Relative changes are expressed as [(x_f-x_i)/x_i × 100%], where x_f corresponds to the values of unit-cell parameters (a, c) and volume (V) at a certain RH level and x_i corresponds to the initial values measured at the beginning of the cycle.

Level No.	Relative Humidity (%)	$\Delta a/a_i$ (%)	$\Delta c/c_i$ (%)	$\Delta V/V_i$ (%)
1	73	0.00000%	0.00000%	0.00000%
2	85	0.26524%	-0.85190%	-0.32525%
3	80	0.22596%	-0.93947%	-0.49131%
4	90	0.31321%	-1.25737%	-0.63785%
5	73	0.02722%	-0.16512%	-0.11078%
6	80	0.12240%	-0.65824%	-0.41490%
7	73	0.01373%	-0.14504%	-0.11764%
8	85	0.34635%	-1.41852%	-0.73447%
9	80	0.26006%	-1.20001%	-0.68547%
10	90	0.61508%	-1.84725%	-0.63614%
11	85	0.57773%	-1.81769%	-0.68000%
12	95	0.65078%	-1.88228%	-0.60109%
13	85	0.65356%	-1.90434%	-0.61792%
14	95	0.65097%	-1.98363%	-0.70339%
15	80	0.66822%	-1.81182%	-0.49523%
16	85	0.55252%	-1.69822%	-0.60898%
17	73	0.13503%	-0.69559%	-0.42722%

Table S23. Refined unit-cell parameters and volume of tetragonal HEWL (space group $P4_32_12$) **for the 5th cycle of the 5th series**, as obtained via Pawley analysis. Data were collected using a laboratory X-ray powder diffractometer X'Pert Pro (Malvern Panalytical) equipped with an MHC-trans humidity and temperature chamber (Anton Paar), at a fixed temperature of 294.15 K [λ = 1.540585(3) Å].

Level No.	Relative Humidity (%)	Space Group	Unit-cell parameters				R_{wp} (%)	χ^2
			$a = b$ (Å)	c (Å)	$\alpha = \beta = \gamma$ (°)	V (×10^5 Å^3)		
1	95	$P4_32_12$	78.90(2)	38.27(3)	90	2.382(2)	7.9265	1.91826
2	90	$P4_32_12$	78.80(2)	38.32(2)	90	2.380(2)	7.4998	2.29471
3	85	$P4_32_12$	78.70(2)	38.37(2)	90	2.377(2)	7.1226	2.17622
4	80	$P4_32_12$	78.60(2)	38.42(3)	90	2.374(2)	6.7021	2.03848
5	75	$P4_32_12$	78.485(8)	38.47(1)	90	2.3696(7)	5.8652	1.74263
6	80	$P4_32_12$	78.585(9)	38.41(1)	90	2.3720(7)	5.8608	1.75512
7	85	$P4_32_12$	78.68(1)	38.36(1)	90	2.3749(9)	5.8863	1.75605
8	90	$P4_32_12$	78.78(1)	38.30(1)	90	2.3766(7)	6.1268	1.83229
9	95	$P4_32_12$	78.86(1)	38.24(1)	90	2.3777(9)	6.8477	2.04481

Table S24. Percentage variations of the unit-cell parameters and volume of tetragonal HEWL **for the 5th cycle of the 5th series**. Relative changes are expressed as [(x_f-x_i)/x_i × 100%], where x_f corresponds to the values of unit-cell parameters (a, c) and volume (V) at a certain RH level and x_i corresponds to the initial values measured at the beginning of the cycle.

Level No.	Relative Humidity (%)	$\Delta a/a_i$ (%)	$\Delta c/c_i$ (%)	$\Delta V/V_i$ (%)
1	95	0.00000%	0.00000%	0.00000%
2	90	-0.11908%	0.13330%	-0.10504%
3	85	-0.24366%	0.26396%	-0.22407%
4	80	-0.36951%	0.39510%	-0.34548%
5	75	-0.52045%	0.52644%	-0.51724%
6	80	-0.39365%	0.36967%	-0.41899%
7	85	-0.26671%	0.23953%	-0.29447%
8	90	-0.15214%	0.08310%	-0.22121%
9	95	-0.04996%	-0.07437%	-0.17423%

Table S25. Refined unit-cell parameters and volume of tetragonal HEWL (space group $P4_32_12$) **for the 6th cycle of the 5th series**, as obtained via Pawley analysis. Data were collected using a laboratory X-ray powder diffractometer X'Pert Pro (Malvern Panalytical) equipped with an MHC-trans humidity and temperature chamber (Anton Paar), at a fixed temperature of 294.15 K [λ = 1.540585(3) Å].

Level No.	Relative Humidity (%)	Space Group	Unit-cell parameters				R_{wp} (%)	χ^2
			$a = b$ (Å)	c (Å)	$\alpha = \beta = \gamma$ (°)	V (×10⁵ Å³)		
1	95	$P4_32_12$	78.835(8)	38.256(9)	90	2.3776(6)	5.2699	1.64013
2	85	$P4_32_12$	78.789(5)	38.292(4)	90	2.3771(3)	5.7532	1.75147
3	75	$P4_32_12$	78.651(6)	38.352(8)	90	2.3725(5)	5.2711	1.61392
4	70	$P4_32_12$	78.60(1)	38.38(1)	90	2.3711(9)	5.0455	1.53376
5	69	$P4_32_12$	78.57(6)	38.40(6)	90	2.370(4)	4.7016	1.24388
6	67	$P4_32_12$	78.5(1)	38.4(1)	90	2.369(8)	4.6218	1.21219
7	65	$P4_32_12$	78.4(2)	38.4(1)	90	2.36(1)	4.8081	0.96272
18	70	$P4_32_12$	78.49(7)	38.36(7)	90	2.363(5)	4.4314	1.13214
19	75	$P4_32_12$	78.54(1)	38.33(2)	90	2.364(1)	4.5799	1.17352
20	80	$P4_32_12$	78.589(5)	38.299(5)	90	2.3655(3)	4.7336	1.24442
21	85	$P4_32_12$	78.644(9)	38.261(7)	90	2.3664(5)	4.8624	1.28715
22	90	$P4_32_12$	78.72(1)	38.23(1)	90	2.3687(9)	4.9396	1.49713
23	95	$P4_32_12$	78.84(1)	38.20(1)	90	2.3742(9)	4.9311	1.50288

Table S26. Percentage variations of the unit-cell parameters and volume of tetragonal HEWL **for the 6th cycle of the 5th series**. Relative changes are expressed as [$(x_f-x_i)/x_i$ × 100%], where x_f corresponds to the values of unit-cell parameters (a, c) and volume (V) at a certain RH level and x_i corresponds to the initial values measured at the beginning of the cycle.

Level No.	Relative Humidity (%)	$\Delta a/a_i$ (%)	$\Delta c/c_i$ (%)	$\Delta V/V_i$ (%)
1	95	0.00000%	0.00000%	0.00000%
2	85	-0.05741%	0.09243%	-0.02247%
3	75	-0.23274%	0.25050%	-0.21564%
4	70	-0.29766%	0.32434%	-0.27200%
5	69	-0.34024%	0.37994%	-0.30199%
6	67	-0.43666%	0.39787%	-0.47704%
7	65	-0.59990%	0.41614%	-0.78504%
18	70	-0.43754%	0.27264%	-0.60292%
19	75	-0.37355%	0.19385%	-0.55333%
20	80	-0.31117%	0.11331%	-0.50875%
21	85	-0.24128%	0.01289%	-0.46913%
22	90	-0.14892%	-0.07748%	-0.37488%
23	95	0.00310%	-0.15043%	-0.14426%

Table S27. Refined unit-cell parameters and volume of tetragonal HEWL (space group $P4_32_12$) **for the 7th cycle of the 5th series**, as obtained via Pawley analysis. Data were collected using a laboratory X-ray powder diffractometer X'Pert Pro (Malvern Panalytical) equipped with an MHC-trans humidity and temperature chamber (Anton Paar), at a fixed temperature of 294.15 K [λ = 1.540585(3) Å].

Level No.	Relative Humidity (%)	Space Group	Unit-cell parameters				R_{wp} (%)	χ^2
			$a = b$ (Å)	c (Å)	$\alpha = \beta = \gamma$ (°)	V (×10^5 Å^3)		
1	95	$P4_32_12$	78.86(1)	38.276(9)	90	2.3801(7)	6.3991	2.02202
2	85	$P4_32_12$	78.808(5)	38.318(5)	90	2.3798(3)	6.2779	1.92898
3	75	$P4_32_12$	78.68(2)	38.35(3)	90	2.374(2)	5.2830	1.62768
4	65	$P4_32_12$	78.54(4)	38.42(6)	90	2.370(4)	4.4937	1.21794
20	70	$P4_32_12$	78.5(2)	38.5(1)	90	2.369(9)	4.7183	1.46921
21	80	$P4_32_12$	78.60(1)	38.35(2)	90	2.369(1)	5.2450	1.63265
22	90	$P4_32_12$	78.710(7)	38.314(7)	90	2.3736(4)	6.1297	1.56199
23	95	$P4_32_12$	78.81(1)	38.26(1)	90	2.3766(8)	6.3366	1.83760

Table S28. Percentage variations of the unit-cell parameters and volume of tetragonal HEWL **for the 7th cycle of the 5th series**. Relative changes are expressed as [$(x_f - x_i)/x_i \times 100\%$], where x_f corresponds to the values of unit-cell parameters (a, c) and volume (V) at a certain RH level and x_i corresponds to the initial values measured at the beginning of the cycle.

Level No.	Relative Humidity (%)	$\Delta a/a_i$ (%)	$\Delta c/c_i$ (%)	$\Delta V/V_i$ (%)
1	95	0.00000%	0.00000%	0.00000%
2	85	-0.06114%	0.11059%	-0.01181%
3	75	-0.22407%	0.19806%	-0.25045%
4	65	-0.40190%	0.37071%	-0.43444%
20	70	-0.45662%	0.46233%	-0.45301%
21	80	-0.32455%	0.20201%	-0.44738%
22	90	-0.18568%	0.09931%	-0.27209%
23	95	-0.05440%	-0.03890%	-0.14764%

Table S29. Summary of XRPD data collection parameters.

Parameter	Value
Instrument	X'Pert Pro (Malvern Panalytical)
Goniometer	PW3050/60 (Theta/Theta)
Radiation	Cu Kα (λ = 1.5406 Å)
Tube settings	45 kV, 40 mA
Scan mode	Continuous, 2θ scan
Angular range	1.2–30° (2θ)
Counting time	29 s (continuous scan normalization)
Measurement duration	~10 min per scan
Geometry	Transmission
Detector	PIXcel1D
Divergence slit	Fixed 1/4°
Temperature	294.1 ± 0.1 K (~21 °C)
Humidity control	5–95% RH (MHC-trans chamber)
